# Stakeholder involvement in systematic reviews: a scoping review

**DOI:** 10.1186/s13643-018-0852-0

**Published:** 2018-11-24

**Authors:** Alex Pollock, Pauline Campbell, Caroline Struthers, Anneliese Synnot, Jack Nunn, Sophie Hill, Heather Goodare, Jacqui Morris, Chris Watts, Richard Morley

**Affiliations:** 1 Nursing Midwifery and Allied Health Professions (NMAHP) Research Unit, Glasgow Caledonian University, Cowcaddens Road, Glasgow, G4 0BA UK; 2 EQUATOR Network, Centre for Statistics in Medicine, NDORMS, University of Oxford, Botnar Research Centre, Windmill Road, Oxford, OX3 7LD UK; 3 Cochrane Consumers and Communication, Centre for Health Communication and Participation, School of Psychology and Public Health, La Trobe University, Kingsbury Drive, Bundoora, Victoria 3086 Australia; 4 Cochrane Australia, School of Public Health and Preventive Medicine, Monash University, L4, 551 St Kilda Road, Melbourne, Victoria 3004 Australia; 5 Centre for Health Communication and Participation, School of Psychology and Public Health, La Trobe University, Kingsbury Drive, Bundoora, Victoria 3086 Australia; 6 Edinburgh, UK; 7 School of Nursing and Health Sciences, University of Dundee, 11 Airlie Place, Dundee, DD1 4HJ UK; 8 Cochrane Learning and Support Department, Cochrane Central Executive, St Albans House, 57-59 Haymarket, London, SW1Y 4QX UK; 9 Cochrane Consumer Network, St Albans House, 57-59 Haymarket, London, SW1Y 4QX UK

**Keywords:** Systematic review, Scoping review, Involvement, Stakeholder, Patient, Public, Consumer

## Abstract

**Background:**

There is increasing recognition that it is good practice to involve stakeholders (meaning patients, the public, health professionals and others) in systematic reviews, but limited evidence about how best to do this. We aimed to document the evidence-base relating to stakeholder involvement in systematic reviews and to use this evidence to describe how stakeholders have been involved in systematic reviews.

**Methods:**

We carried out a scoping review, following a published protocol. We searched multiple electronic databases (2010–2016), using a stepwise searching approach, supplemented with hand searching. Two authors independently screened and discussed the first 500 abstracts and, after clarifying selection criteria, screened a further 500. Agreement on screening decisions was 97%, so screening was done by one reviewer only. Pre-planned data extraction was completed, and the comprehensiveness of the description of methods of involvement judged. Additional data extraction was completed for papers judged to have most comprehensive descriptions. Three stakeholder representatives were co-authors for this systematic review.

**Results:**

We included 291 papers in which stakeholders were involved in a systematic review. Thirty percent involved patients and/or carers. Thirty-two percent were from the USA, 26% from the UK and 10% from Canada. Ten percent (32 reviews) were judged to provide a comprehensive description of methods of involving stakeholders. Sixty-nine percent (22/32) personally invited people to be involved; 22% (7/32) advertised opportunities to the general population. Eighty-one percent (26/32) had between 1 and 20 face-to-face meetings, with 83% of these holding ≤ 4 meetings. Meetings lasted 1 h to ½ day. Nineteen percent (6/32) used a Delphi method, most often involving three electronic rounds. Details of ethical approval were reported by 10/32. Expenses were reported to be paid to people involved in 8/32 systematic reviews.

**Discussion/conclusion:**

We identified a relatively large number (291) of papers reporting stakeholder involvement in systematic reviews, but the quality of reporting was generally very poor. Information from a subset of papers judged to provide the best descriptions of stakeholder involvement in systematic reviews provide examples of different ways in which stakeholders have been involved in systematic reviews. These examples arguably currently provide the best available information to inform and guide decisions around the planning of stakeholder involvement within future systematic reviews. This evidence has been used to develop online learning resources.

**Systematic review registration:**

The protocol for this systematic review was published on 21 April 2017. Publication reference: Pollock A, Campbell P, Struthers C, Synnot A, Nunn J, Hill S, Goodare H, Watts C, Morley R: Stakeholder involvement in systematic reviews: a protocol for a systematic review of methods, outcomes and effects. Research Involvement and Engagement 2017, 3:9. https://doi.org/10.1186/s40900-017-0060-4.

**Electronic supplementary material:**

The online version of this article (10.1186/s13643-018-0852-0) contains supplementary material, which is available to authorized users.

## Background

The concept of active involvement in research of people with a healthcare condition, their families, friends and carers, was founded on the principle that people affected by the condition have a moral right to contribute to decisions about what research is undertaken and in what way [1–3]. The active involvement of other stakeholders (meaning patients, the public, health professionals, health decision makers and funders) grew from a desire to address the lack of real-world relevance of research and to ensure more effective implementation of research findings into practice [4, 5]. It is now widely accepted in many parts of the world that the active involvement of many of these groups (that we collectively refer to as ‘stakeholders’) is beneficial to the quality, relevance and impact of health research [2, 3]. Accordingly, many funding bodies, including government and charities, now mandate that researchers actively involve patients and the public in their research, including systematic reviews [6–9], although there is evidence of international variation in the extent to which patients and the public are involved [10].

Systematic reviews aim to inform and support the delivery of evidence-based practice, by finding and bringing together, in an explicit and transparent way, all the research evidence that addresses a particular topic or healthcare question. Stakeholder involvement within systematic reviews has been proposed as a way to enhance the actual and perceived usefulness of synthesised research evidence, addressing barriers to the uptake of evidence into practice [11]. In this paper, we define (based on a number of published definitions, e.g. [1, 12, 13]) ‘active stakeholder involvement’ as the contribution of people who are not researchers throughout the process of production and dissemination of a systemic review, including the planning and conduct of an individual systematic review. While there are a number of examples of active stakeholder involvement in systematic reviews, the approaches to, and extent of, involvement have varied considerably [14–16] and synthesised evidence and resources to guide practice is lacking. As well as active involvement within individual systematic reviews, stakeholders may also get involved at the level of organisations which commission or carry out systematic reviews. A recent review explored examples of consumer involvement within organisations (such as Cochrane) that support production of systematic reviews [17], but evidence relating to relevant activities and roles of individual researchers and how they may involve stakeholders in their reviews remains scant [18].

As part of a wider project to provide guidance to researchers about how to involve stakeholders in systematic reviews [19], we undertook a mixed-method evidence synthesis, first completing a scoping review to create a broad map of evidence relating to stakeholder involvement in systematic reviews, followed by two contingent syntheses [20]. Here, we report the results of the scoping review. The aims of this paper are therefore to:

1. Document the evidence-base relating to stakeholder involvement in systematic reviews
2. Use this evidence to describe key features of how stakeholders have been involved in systematic reviews

## Methods

### Design

We carried out a scoping review, following a protocol [20]. We followed the methodological steps outlined by Arksey and O’Malley [21] and used an iterative team approach, with regular team meetings to discuss progress and reach consensus on next steps, to ensure clarity of purpose and balance between breadth and comprehensiveness of the review [21–23]. Protocol deviations, with justifications, are described in Additional file 1.

### Search strategy

We implemented a stepwise approach [24] to promote efficient identification of up-to-date literature, balancing the expected large volume of literature with available time and resources. Details of this approach, including pre-agreed criteria and contingencies to inform decisions relating to the extent of the searches, have previously been described [20]; below we report the actual steps of searching and brief justification for these steps.

We used a comprehensive search strategy, adapted for each database (see Additional file 2). In step 1, we searched a comprehensive set of databases (CENTRAL (CDSR, DARE, HTA, Cochrane Methodology Register), Embase (Ovid), MEDLINE (Ovid), CINAHL (EBSCO), AMED, Joanna Briggs Database and ProQuest Dissertations and Theses (handsearched)), within a narrow time period (from 01 January 2014 to 09 April 2016). The aim of step 1 was to, in an efficient way, identify the databases most likely to include relevant papers. In step 2, we searched a more limited set of databases (Embase, MEDLINE, CINAHL and HTA) for a longer time period (01 January 2010 to 31 December 2013) with the aim of exploring whether there was justification for extending the search beyond 2010.

Searching and application of inclusion criteria was applied to each step prior to progression to the next step. For step 1, we noted the source database (or databases) of each identified record, and the databases from which the greatest number of included papers were identified. The results of these explorations were discussed and review team consensus reached on which databases to include in step 2. After step 2, the review team explored the publication dates of records meeting our inclusion criteria. The majority of papers meeting our inclusion criteria (63%) were published in either 2014 or 2015 (see Additional file 3). The sharp drop in numbers of included papers from 2014 to 2013 and relatively stable number of included papers between 2013 and 2010 were key factors in the team decision not to extend electronic searching to before 2010.

Additional sources we searched include the reference lists of recent relevant reports and reviews (e.g. [6, 17, 25]), the reference lists of all included studies and articles published in the journal *Research Involvement and Engagement*. To identify unpublished reports, we contacted authors of published papers and promoted this review via social media.

### Selection criteria

Selection criteria for inclusion were purposefully wide. We included any paper, published or unpublished, regardless of study design, including commentaries, letters and expert opinion, which investigated, reported or discussed any aspect of stakeholder involvement in a systematic review. We anticipated that we would include (but would not be limited to) evidence such as published systematic reviews which reported involvement; reports of methods of involvement in an individual systematic review; studies quantitatively or qualitatively evaluating involvement in individual systematic reviews; and opinions, commentary and discussion relating to involvement in systematic reviews.

We excluded papers that focussed on stakeholder involvement in the generation of research priorities (unless they were specifically generating questions for a systematic review) and in both research more broadly, and guideline development, unless there was an explicit mention of involvement in systematic reviews. Systematic reviews that focussed on synthesising the evidence related to stakeholder involvement in primary research were also excluded. We excluded titles without abstracts and review protocols; this was a pragmatic decision made in light of the high volume of search results.

#### Definition of key terms

We used the following operational definitions, pre-stated in the protocol [20], to support the application of the selection criteria:

- Stakeholder—*any person who would be a knowledge user of research but whose primary role is not directly in research*. Potential stakeholders include a broad range of people, including those who are actual or potential recipients of health or social care, where this may include patients, carers and family members, or people interested in remaining healthy who are seeking information about a health condition or treatment for personal use [26]; members of organisations that represent people who use services; people with a professional role in health and social care; policy makers and managers. We documented the types of people involved within any evidence included in this review, highlighting where this included patients, carers and family members, and where this included other stakeholders only.
- Systematic review—*a research process in which literature relevant to a stated question is identified and brought together (synthesised) using explicit methods* [27], *including reporting of inclusion/exclusion criteria, search methods and details of included studies*. We accepted systematic reviews regardless of the type of evidence synthesised (i.e. quantitative, qualitative, mixed-methods) and the type of question addressed (e.g. intervention effectiveness, diagnostic test accuracy, patient experiences).
- Involvement in a systematic review—*any role or contribution of stakeholders toward the development of a review protocol, completion of any of the stages of a systematic review or dissemination of the findings of a review*.

### Methods of applying selection criteria

One review author (PC) ran the search strategy and excluded any obviously irrelevant titles. Two reviewers (PC, AP) independently reviewed the abstracts and applied selection criteria to the first 500 records; agreement was explored and a full team discussion held to clarify the selection criteria. This clarification led to a number of post hoc exclusion criteria (described above under selection criteria and within Additional file 1). Subsequently, we agreed that two independent review authors (PC, AP) should review a further 500 records using the clarified criteria and that if agreement between independent reviewers was greater than 95% when using these refined criteria, then subsequent selection of papers would be performed by one reviewer only; this agreement was 97%, and therefore, one reviewer (AP) screened the remaining abstracts. The full papers from abstracts included after the screening process were considered at the data extraction and judgement stages (see below); if a paper was found not to meet the inclusion criteria at this stage, it was excluded.

### Data extraction and synthesis

#### Data extraction

For all included papers, one reviewer (AP) extracted and categorised data into structured tables. Extracted data included bibliographic information, type of paper, stated aim, topic/focus of systematic review, study/review methodology, description of reported involvement, details of people involved, stage in review process at which people involved and any formal research methods used. Retrospective categorisation of data included focus of review and type of evidence synthesised (see Additional file 1, protocol deviations). Details of the operationalisation of these data extraction items are provided in Additional file 4.

#### Judgement of comprehensiveness of description

Our review aim was to describe key features of how stakeholders have been involved in systematic reviews; consequently, we were principally concerned with the comprehensiveness of the description of methods of involvement, rather than appraising the quality of the methods of the reviews. We devised a method for judging the comprehensiveness of the description of the method or approach to involvement, given that there are no standardised tools for such a task. Criteria for categorising the comprehensiveness of the description provided within papers were developed, adapted from Pollock [28]. Initially, two reviewers (AP, CS) assigned these criteria independently for a random sample of 20% of papers identified from step 1 of searching; this was 42 of 210 papers. There was agreement between independent reviewers for 57% (24/42) of the assessed sample. The agreement between reviewers, implications relating to disagreements and perceived risk of bias to the review results are reported in Additional file 5. Following discussion and clarification of criteria (see Additional file 5), it was agreed that one reviewer would assign judgements to the remaining papers, using the following criteria:

- ‘Green’—comprehensive description of one (or more) specific method or approach to the involvement of stakeholders in systematic reviews. Description sufficient to enable replication of methods
- ‘Amber’—a brief or partial description of one (or more) specific method or approach to the involvement of stakeholders in systematic reviews. Description sufficient to enable partial replication of methods
- ‘Red’—few details provided and/or inadequate description of the method or approach of involvement of stakeholders in systematic reviews. Description insufficient to enable any replication of methods

#### Detailed description of methods or approaches to involvement

Additional, more detailed, data extraction was performed for papers that were judged as ‘green’ for comprehensiveness of description. In addition to a narrative description of the methods or approaches to involvement, one reviewer (AP) extracted and tabulated the stated aim of involvement, number and characteristics of people involved, methods of recruitment, format of involvement (e.g. face-to-face meeting, telephone meeting, written consultation, online survey), amount of involvement (number of meetings, number of days involved), details of ethical approval and financial compensation given to stakeholders, evaluation of the involvement and tools used for reporting involvement.

### Stakeholder involvement in this systematic review

One consumer (HG) and two consumer representatives (RM, CS) were members of the project and author team for this systematic review. All contributed to face-to-face discussions which led to the development of the review protocol, and read, commented on and had authorship of the published protocol. All contributed to project teleconferences throughout the review, particularly when making decisions relating to the stepwise search methods. Additionally, CS independently applied judgements of comprehensiveness to a sample of full papers. All three discussed the key findings of this review and contributed as authors to the final manuscript.

## Results

### Results of the search

**Fig. 1:**
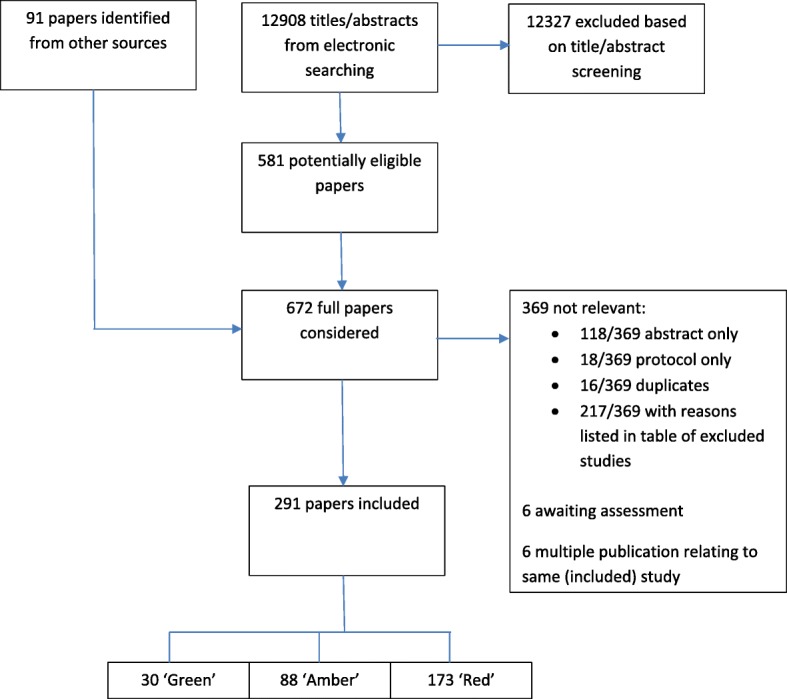
PRISMA flowchart

We screened 12,908 titles and abstracts and applied selection criteria to 672 full papers. Three hundred sixty-nine of these 672 full papers were excluded: 118 as they were abstracts only, 18 as they were protocols, 16 as they were duplicates and 217 as they did not meet our inclusion criteria. Reasons that these 217 did not meet our inclusion criteria are listed in table of excluded studies (Additional file 6); main reasons for exclusion were that the paper was a systematic review but there was no involvement of people (approximately 30%), the paper did not describe or report a systematic review (approximately 25%) or the paper described involvement in research other than a systematic review (approximately 25%). This left 291 papers that met our criteria for inclusion in the scoping review (see Fig. 1).

### Characteristics of included papers

Details of the 291 included papers are provided in the table of included studies (Additional file 7). A brief summary is described below.

#### Type of paper

Thirty-one percent of included papers were published systematic reviews; 54% were reports of a guideline or recommendation in which a systematic review component was described; and 5% were papers specifically describing methods of involving stakeholders in a systematic review.

#### Stakeholders involved

Thirty percent of the included papers involved patients and/or carers within the systematic review process, while 41% involved other stakeholders (e.g. health professionals, academic experts, representatives of patient organisations) but not patients or their family members. In almost one third of the included papers (29%), it was not clear who the stakeholders involved in the review were and whether this included patients and/or carers.

#### Country

One third (31.6%) of papers were from the USA, one quarter (26.1%) from the UK and 10.0% from Canada. Of the remaining papers, 22.7% were from Australia, Netherlands, Germany, Italy, France or Spain, and 9.6% from a further 15 countries with between 1 and 4 papers each (see Table 1).

**Table 1:** Country in which stakeholder involvement took place/country of lead author

	Total		Patients/carers included		No patients/carers included		Unclear	
USA	92	31.6%	18	19.6%	42	45.7%	32	34.8%
UK	76	26.1%	42	55.3%	16	21.1%	18	23.7%
Canada	29	10.0%	3	10.3%	17	58.6%	9	31.0%
Australia	22	7.6%	5	22.7%	7	31.8%	10	45.5%
Netherlands	14	4.8%	8	57.1%	4	28.6%	2	14.3%
Germany	12	4.1%	2	16.7%	8	66.7%	2	16.7%
Italy	7	2.4%	1	14.3%	4	57.1%	2	28.6%
France	6	2.1%	1	16.7%	3	50.0%	2	33.3%
Spain	5	1.7%	3	60.0%	1	20.0%	1	20.0%
Other (countries with < 5 papers*)	28	9.6%	5	17.9%	17	60.7%	6	21.4%

*Austria, China, Japan, Saudi Arabia, Switzerland, Belgium, Denmark, Norway, Brazil, Chile, Columbia, Iran, Korea, New Zealand, Taiwan

#### Stage of the review process

In almost half of the papers (47.8%), the stage of the review process at which stakeholders were involved was unclear. In just over one quarter (27.5%), stakeholders were involved in interpreting the results after the evidence had been synthesised. In around one fifth (22.3%), stakeholders were involved either throughout the whole review process or during one or more stages of review completion (see Table 2).

**Table 2:** Stage of the review process in which stakeholders were involved

	Total*		Patients/carers involved		No patients/carers involved		Unclear	
Setting scope/review questions	25	8.59%	8	32.00%	15	60.00%	2	8.00%
Interpreting results after review completion	80	27.49%	27	33.75%	39	48.75%	14	17.50%
Throughout/within the review process	65	22.34%	36	55.40%	24	36.90%	5	7.70%
unclear	139	47.77%	22	15.80%	52	37.40%	65	46.80%

*Percentages are calculated as percentage of the 291 papers with involvement at that stage. Total adds up to > 100%, as 18 papers involved stakeholders at both setting scope/review questions and interpreting results after review completion and have therefore been counted within both of these categories

#### Focus of the review

Seventy-one percent of the included systematic reviews were judged to be focussed on one of the International Statistical Classification of Diseases and Related Health Problems 10th Revision (ICD-10) categories (Table 3 and table of included studies (Additional file 7)). Most frequently (10%), this was ‘factors influencing health status and contact with health services’, where reviews covered topics such as the effectiveness or implementation of care pathways for specific population (e.g. paediatrics, geriatrics, emergency care). The specific diseases or health areas covered by the greatest numbers of reviews were mental and behavioural disorders (8.6%), neoplasms (6.9%), diseases of the musculoskeletal system and connective tissue (6.2%) and certain infectious and parasitic diseases (5.5%). Thirteen percent of the reviews which did not fit one of the ICD-10 categories were focussed on a specific intervention, most commonly medical or surgical interventions (8.6%) and public health interventions (5.2%). Ten percent of reviews were focussed on an area of research, rather than a specific health or disease area or intervention; more than half of these (55%, *n* = 29) were focussed on methods of stakeholder involvement or engagement, while the remainder focussed on other areas of research methods, such as methods of statistical tests within primary research. The remaining 7% were unable to be categorised within any of these groups and focussed on, for example, areas such as teaching, data protection and criminal justice.

**Table 3:** Focus of review

	Total	Patients/carers included	No patients/carers included	Unclear
Research	29 10.0%	16 55.2%	7 24.1%	6 20.7%
XXI Factors influencing health status and contact with health services	28 9.6%	9 32.1%	15 53.6%	4 14.3%
V Mental and behavioural disorders	25 8.6%	12 48.0%	4 16.0%	9 36.0%
II Neoplasms	20 6.9%	7 35.0%	7 35.0%	6 30.0%
ICHI Medical and surgical intervention	20 6.9%	1 5.0%	12 60.0%	7 35.0%
XIII Diseases of the musculoskeletal system and connective tissue	18 6.2%	6 33.3%	10 55.6%	2 11.1%
I Certain infectious and parasitic diseases	16 5.5%	4 25.0%	10 62.5%	2 12.5%
ICHI Public health intervention	15 5.2%	3 20.0%	8 53.3%	4 26.7%
XI Diseases of the digestive system	14 4.8%	0 0.0%	6 42.9%	8 57.1%
X Diseases of the respiratory system	13 4.5%	2 15.4%	8 61.5%	3 23.1%
IV Endocrine, nutritional and metabolic diseases	12 4.1%	1 8.3%	6 50.0%	5 41.7%
IX Diseases of the circulatory system	12 4.1%	2 16.7%	4 33.3%	6 50.0%
XII Diseases of the skin and subcutaneous tissue	8 2.7%	3 37.5%	4 50.0%	1 12.5%
XIV Diseases of the genitourinary system	8 2.7%	3 37.5%	3 37.5%	2 25.0%
XV Pregnancy, childbirth and the puerperium	8 2.7%	4 50.0%	2 25.0%	2 25.0%
XIX Injury, poisoning and certain other consequences of external causes	7 2.4%	0 0.0%	3 42.9%	4 57.1%
VI Diseases of the nervous system	5 1.7%	2 40.0%	2 40.0%	1 20.0%
Categories with < 5 papers*	14 4.8%	7 50.0%	1 7.1%	6 42.9%
Other (unable to categorise)	19 6.5%	6 31.6%	7 36.8%	6 31.6%

Note: no papers were categorised as XVI Certain conditions originating in the perinatal period; XVIII Symptoms, signs and abnormal clinical and laboratory findings, not elsewhere classified

*III Diseases of the blood and blood-forming organs and certain disorders involving the immune mechanism; VII Diseases of the eye and adnexa; VIII Diseases of the ear and mastoid process; XVII Congenital malformations, deformations and chromosomal abnormalities; XX External causes of morbidity and mortality; ICHI Functioning intervention

### Comprehensiveness of description of method or approach to involvement

**Fig. 2:**
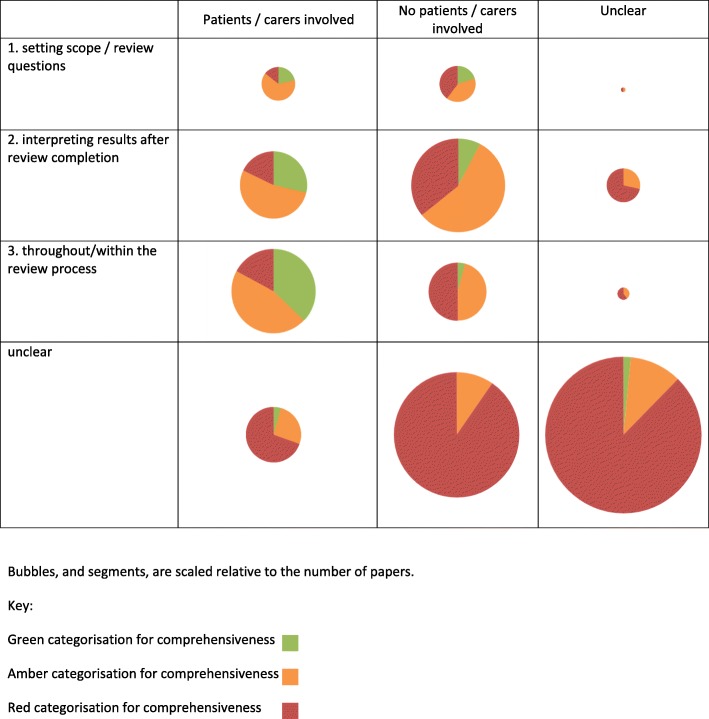
Bubble plot illustrating proportion of papers in which patients/carers were included, the stage of the review process at which people were involved and the comprehensiveness of the description of the method of involvement

Table 4 shows the assigned judgements of the comprehensiveness of the description of the method or approach to involvement. Figure 2 illustrates the proportion of different types of paper which were judged to be ‘green’, ‘amber’ or ‘red’, when patients/carers were involved and at different stages in the review process.

**Table 4:** Comprehensiveness of description of method or approach to involvement

Comprehensiveness	‘Green’	‘Amber’	‘Red’
	Comprehensive description of one (or more) specific method or approach to the involvement in systematic reviews. Description sufficient to enable replication of methods	A brief or partial description of one (or more) specific method or approach to the involvement in systematic reviews. Description sufficient to enable partial replication of methods	Few details provided and/or inadequate description of the method or approach of involvement. Description insufficient to enable any replication of methods
Total	30	88	173
Were patients/consumers involved?			
Yes	24	37	27
No	5	38	76
Unclear	1	13	70
Stage at which there was involvement*			
Setting scope/review questions	7	11	7
Interpreting results after review completion	10	41	29
Throughout/within the review process	15	29	21
Unclear	1	18	120

*18 papers involved stakeholders at both setting scope/review questions and interpreting results after review completion; 3 were judged as ‘green’, 11 as ‘amber’ and 4 as ‘red’

In total, 59% of the included papers were judged to provide few or inadequate details (‘red’), with only 10% judged to provide a comprehensive description of one, or more, method or approach to involvement (‘green’).

### Detailed description of methods or approaches to involvement

The 30 papers which were judged as providing a comprehensive (‘green’) description of their methods or approaches to involvement included 14 ‘methods’ papers describing an experience of stakeholder involvement in one (or more) systematic review [25, 29–41]; 11 systematic reviews in which the stakeholder involvement was concurrently described [42–52]; 2 guidelines or clinical recommendations, in which the involvement in the systematic review component was described [53, 54]; and 1 paper which described the development of a tool to report stakeholder involvement [55]. Two of the papers each described two different systematic reviews [37, 55], meaning that there are a total of 32 systematic review described. Table 5 summarises the key characteristics of these 32 systematic reviews, and Table 6 summarises the data relating to stakeholder involvement and a brief narrative summary of key features is provided below.

**Table 5:** Key characteristics of ‘green’ systematic reviews

Review	Geographical location	Type of paper	Review aim/focus	Focus of review*	Type of evidence synthesised**
Bayliss et al. [29]	Europe (UK, Sweden, Estonia, Romania)	Description of methods of involvement (describes results of questionnaire-based evaluation of the involvement)	Qualitative meta-synthesis; perceptions of predictive testing for those at risk of developing a chronic inflammatory disease	XIII Diseases of the musculoskeletal system and connective tissue	Qualitative
Boelens et al. [30]	Europe	Description of methods of involvement (describes the consensus process to develop statements and recommendations to include in a patient summary)	Patient summary of consensus for colon and rectal cancer care	II Neoplasms	Unclear
Bond et al. [53]	Australia	Report of a guideline/recommendation	Guidelines; assisting Australians with mental health problems and financial difficulties.	V Mental and behavioural disorders	Mixed
Braye and Preston-Shoot [31]	UK	Description of methods of involvement (researcher’s experiences of involving stakeholders)	Systematic review; learning, teaching and assessment of law in social work education	Other	Mixed
Bunn et al. [32]	UK	Description of methods of involvement (method to contextualise findings of a review)	Systematic review of qualitative studies; patient and carer experiences of diagnosis and treatment of dementia	V Mental and behavioural disorders	Qualitative
Concannon et al. [42]	USA	Systematic review	Systematic review; methods of stakeholder engagement in comparative effectiveness research and patient-centred outcomes research	Research	Mixed
Coon et al. [63]	UK	Description of methods of involvement (describe and discuss methods of involvement in 4 reviews)	Four systematic reviews; nonpharmacological interventions for ADHD used in school settings	V Mental and behavioural disorders	Mixed
Edwards et al. [43]	UK	Systematic review	Identification, assessment and management of risk in young people with complex mental health needs entering, using and exiting inpatient child and adolescent mental health services in the UK	V Mental and behavioural disorders	Mixed
Harris et al. [25]	UK	Description of methods of involvement (describe and discuss methods of involvement in a realist review)	Realist review; community-based peer support	ICHI Functioning intervention	Mixed
Hayden et al. [44]	Canada	Systematic review	Rapid knowledge synthesis; Collaborative Emergency Centres (CECs)	XXI Factors influencing health status and contact with health services	Unclear
Higginson et al. [45]	UK	Systematic review	Systematic review; design and conduct of research on end of life care (EoLC)	XXI Factors influencing health status and contact with health services	Mixed
Hyde et al. [34]	UK	Description of methods of involvement	Systematic review and narrative synthesis; factors affecting shared decision-making around prescribing analgesia for musculoskeletal pain in primary care consultations	XIII Diseases of the musculoskeletal system and connective tissue	Mixed
Jamal et al. [35]	UK	Description of methods of involvement	Systematic review; effects of schools and school environment interventions on children and young people’s health	XXI Factors influencing health status and contact with health services	Mixed
Liabo [46]	UK	Description of methods of involvement	Systematic review; effectiveness of interventions that aim to support looked after children in school	XXI Factors influencing health status and contact with health services	Mixed
Liu et al. [47]	UK	Systematic review	Mixed-methods systematic review; to identify, appraise and interpret research on the approaches employed to maximise the cross-cultural appropriateness and effectiveness of health promotion interventions for smoking cessation, increasing physical activity and improving healthy eating for African-, Chinese- and South Asian-origin populations	ICHI Public health intervention	Mixed
Martin et al. [36]	UK	Description of methods of involvement	Systematic review; attitudes and preferences toward population screening for dementia	V Mental and behavioural disorders	Mixed
McConachie et al. [48]	UK	Systematic review	Systematic review; measurement properties of tools used to measure progress and outcomes in children with autistic spectrum disorder (ASD) up to the age of 6 years	V Mental and behavioural disorders	Quantitative
McCusker et al. [49]	Canada	Systematic review	Systematic review; collaborative mental health care for depression	V Mental and behavioural disorders	Mixed
McGinn et al. [56]	Canada	Systematic review	Systematic review; users’ perspectives of the factors influencing electronic health record (EHR) implementation	XXI Factors influencing health status and contact with health services	Quantitative
Morgan et al. [58]	UK	Systematic review	Systematic review (quantitative and qualitative evidence); incentive mechanisms of action for smoking cessation in pregnancy and breastfeeding	XV Pregnancy, childbirth and the puerperium	Mixed
Oliver et al. [37] (‘correlational’ review)	UK	Description of methods of involvement	Systematic review of observational studies (a ‘correlational’ review); to explore the quantitative relationship between childhood obesity and educational attainment	ICHI Public health intervention	Quantitative
Oliver et al. [37] (‘views’ review)	UK	Description of methods of involvement	Systematic review of interview-based and questionnaire-based research (‘views review’); children’s perspectives of body size	ICHI Public health intervention	Qualitative
Oosterkamp et al. [54]	Netherlands	Report of a guideline/recommendation	Systematic review; strategies to prevent WSL (white spot lesions) during orthodontic treatment with fixed appliances	XI Diseases of the digestive system	Quantitative
Pearson et al. [50]	UK	Systematic review (description of involvement in supplementary file)	Realist review; conditions and actions which lead to the successful implementation of health promotion programmes in schools	ICHI Public health intervention	Mixed
Pollock et al. [38]	UK	Description of methods of involvement	Cochrane review; physical rehabilitation approaches for the recovery of function and mobility in people with stroke	IX Diseases of the circulatory system	Quantitative
Rees et al. [51]	UK	Systematic review	Systematic review; barriers to, or facilitators of, HIV-related sexual health for men who have sex with men (MSM) and MSM’s perceptions and experiences of sexual health in the light of HIV	I Certain infectious and parasitic diseases	Mixed
Saan et al. [55] (review 1)	Netherlands	Other (development of reporting tool)	Qualitative evidence synthesis; needs of victims of crime with regard to helpful and unhelpful reactions of their social network including volunteer services	Other	Qualitative
Saan et al. [55] (review 2)	Netherlands	Other (development of reporting tool)	Systematic review; concepts, determinants and outcome measures used to evaluate self-management support	Other	Unclear
Serrano-Anguilar et al. [39]	Spain	Description of methods of involvement	Systematic review; effectiveness and safety of treatment alternatives for patients with degenerative ataxias (DA)	VI Diseases of the nervous system	Unclear
Smith et al. [52]	UK	Systematic review	‘Multi-methods review’; service user involvement in nursing, midwifery and health visiting research	Research	Mixed
Stewart and Oliver [40]	UK	Description of methods of involvement	Systematic review of reviews; communication with parents about newborn bloodspot screening	XV Pregnancy, childbirth and the puerperium	Mixed
Vale et al. [41]	UK	Description of methods of involvement	Cochrane review; chemoradiotherapy for cervical cancer	II Neoplasms	Quantitative

Note that within this table there are 32 reviews described, as 2 of the 30 included papers describe 2 reviews, and data has been extracted separately for these at this stage (Oliver et al. [37] ‘correlational’ review and ‘views’ review; Saan et al. [55] review 1 and review 2)

*Focus of review categorised as either: Health/disease of focus according to ICD-10 categories; OR, if review was not focused on a specific health topic/disease, then review was categorised as either: Medical or surgical intervention, Public health intervention, Functioning intervention, Research, or Other

**Type of evidence synthesised categorised as qualitative, quantitative, mixed (i.e. both qualitative and quantitative) or unclear

**Table 6:** Summary of data relating to stakeholder involvement in ‘green’ systematic reviews

Review	Description of involvement	People involved	Method of involvement	Formal research methods	Ethical approval?	Financial compensation (or alternative) for people involved?
Bayliss et al. [29]	‘Patient research partners’ contributed via teleconference calls, written (email) and one face-to-face meeting. Teleconferences: during completion of searches and paper selection, to input into search By email: 3 volunteers helped with coding framework to coproduce themes with researchers Meeting: invited to attend a 90-min face-to-face focus session at an annual meeting	Women with rheumatoid arthritis (*n* = 8); all with understanding of the research process	‘Patient research partners’—teleconferences, emails and invitation to one 90-min meeting	–	No information provided	No information provided
Boelens et al. [30]	Experts participated in three web-based online voting rounds, discussion and lectures at a conference. Patients and patient representatives were involved in the Delphi and had a role in developing a patient summary and future testing and implementation of this patient version. Drafts of the patient summary were circulated by email, and comments invited.	‘Experts’ (professionals). People who were colon or rectal cancer survivors and representatives of this group.	Delphi approach (online, 3 rounds) ‘In the Delphi online voting process, 32 delegates were able to vote. Patient representatives had one vote. It is not known how many patients attended the meeting.’	Delphi approach	No information provided	No information provided
Bond et al. [53]	Statements arising from a systematic review were used to form a questionnaire that was administered to the expert panels via SurveyMonkey. The panel members were asked to rate each of the statements, using a 5-point scale, according to whether or not they thought the statement should be included in the guidelines.	Five expert panels: financial counsellors, financial institution staff, mental health professionals, mental health consumer advocates and carer advocates. All panellists had to be 18 years or older, living in Australia, and have either professional or personal experience with mental health problems and financial difficulties.	Delphi approach (online, 3 rounds) (340 participants invited; 214 completed round 1, 170 completed round 2, 162 completed round 3)	Delphi approach	Ethical approval granted.	No information provided
Braye and Preston-Shoot [31]	The first meeting aimed to seek views on the content and process of the study, finalising the research questions and concluding the protocol, and to explore and consider participants perspectives around the topic. The second meeting aimed to present and consider emerging findings and agree recommendations for the final report, and to consider the broader implications.	Service users and carers (*n* = 15), professionals (*n* = 16)	Two face-to-face meetings. Information was sent out prior to meetings, introductory presentation.	–	No information provided	In addition to researcher time to undertake these negotiations, money was also set aside to meet the costs of travel and special transport, accommodation and fees related to attendance........ . It was self-evident here that individuals, or their organisations, should be paid for their time, immediately in cash when required.
Bunn et al. [32]	Focus groups and interviews with key stakeholders. Involvement occurred after completion of the systematic review, in order to confirm the key themes from the review.	27 participants (three people with dementia, 12 carers, six dementia service providers and five older people without dementia). Purposive sampling approach to recruitment.	Four focus groups and three interviews	Focus groups, interviews	Ethical approval was obtained from NRES Committee East of England. REC reference 10/H0302/19.	Participants were given a £10 voucher in appreciation of their time, and their travel expenses were reimbursed.
Concannon et al. [42]	Two meetings at different stages in the review process. Meeting 1 aimed at confirming the research questions and study design. Meeting 2 to review preliminary results. Group members also participated by email and phone, commenting on tables, figures and manuscript drafts, and were asked to assist in dissemination. In addition, 3 people (2 patients) contributed to review the planning stages.	Consumers, professionals, researchers (*n* = 7)	Two face-to-face meetings. Email and phone communication throughout the review.	–	No information provided	No information provided
Coon et al. [63]	An Expert Advisory group—involved throughout the project, including commenting on the protocol, editing draft chapters and responding to ad hoc questions. In addition, a series of four events were held: Event 1 aimed to share information and explore experiences. Events 2 and 3 aimed to explore interim findings of the review. Event 4 disseminated findings.	Expert advisory group—academics, charity representatives, professionals. Four events: Event 1 (*n* = 15), parents, carers, professionals, researchers. Event 2 (*n* = 20), professionals. Event 3 (*n* = 25), parents and young people. Event 4 (*n* = 60), parents, professionals, policy makers.	Expert advisory group. Four events.	–	No information provided	No information provided
Edwards et al. [43]	Individual interviews—aimed at identifying topics for review to focus on. Meeting—descriptive maps from initial scoping review/mapping exercise were presented. Informed by the principles of nominal group technique, participants generated independent lists of important topics, which were collated and displayed. Individual participants then ranked, in writing, their personal priorities from the list.	Individual interviews (*n* = 6), with young people (patients) or their carers. Face-to-face meeting—health professionals, young people, charity representative (*n* = 7), plus research team (*n* = 7)	Two methods: 1) Individual interviews 2) Face-to-face meeting	Consensus decision-making techniques: nominal group technique	No information provided	No information provided
Harris et al. [25]	Five ‘cross-organisation’ events and seven ‘within-organisation’ events. Also email discussions and opportunistic contact with researchers. Recruitment to the advisory network took place throughout the review, and different individuals had different levels of involvement, and at different stages. Some members contributed on multiple occasions and others only on a single occasion.	‘Advisory network’—salaried workers, health trainers, volunteer health champions and programme coordinators with expertise in using peer support, and people who had originally received support before going on to become a peer support worker.	Total of 12 meetings (approximately 240 face-to-face contacts with around 120 participants) Format—various, including use of notes, flip charts, audio recording, post-it notes	Participatory approaches	No information provided	No information provided
Hayden et al. [44]	Workshop 1 = The goals of the stakeholder workshop were to discuss methods for evidence synthesis in general; to discuss the objectives and approach of our specific project; to refine definitions and priorities within the project; and to discuss key findings, key messages, and dissemination plans. Workshop 2 = Key messages and the data tables were discussed at a second stakeholder workshop.	Selected local stakeholders (*n* = 19) (professionals; no consumers involved)	Group meetings (2) (half day). Voting for prioritisation. Format—activities and small group discussions to engage attendees, to encourage discussion, and to reach consensus.	–	No information provided	No information provided
Higginson et al. [45]	‘Transparent expert consultations (TEC)’ involving consensus methods of nominal group and online voting, and stakeholder workshops.	Panel of experts in trials, quantitative, qualitative and mixed-method research, within and outside palliative care, patients/consumers, service providers, clinicians, commissioners, national policy makers and voluntary sector representatives	Transparent Expert Consultation (5 meetings). 3 workshops—2 with patients/consumers, 1 with clinicians/policy makers	Transparent Expert Consultation (incorporates NGT)	Ethics The research ethics committee of the University of Manchester (reference number 10328) approved the TEC component of MORECare. All TEC participants gave written consent.	No information provided
Hyde et al. [34]	Three meetings at different stages in the review process. Meeting 1 took place during the protocol design stage and aimed at refining the scope of the review. Meeting 2 took place at the review preliminary findings stage, aimed at interpreting the results and planning the dissemination. Meeting 3 took place at the writing up stage, aimed at agreeing final results and planning how to share results.	Patients (*n* = 5), members of an established patient research user group (patients with musculoskeletal conditions)	Each face-to-face meeting was 3 h. Format of meetings were a mixture of presentations with discussion, and using ‘small group techniques’	–	No information provided	No information provided
Jamal et al. [35]	Meeting 1—aimed at informing developed of review questions. Discussion around key terms and perspectives of potential interventions. Researchers used data from meeting to generate topics. Meeting 2—aimed at prioritising topics. Discussion around list of topics generated from meeting 1, followed by voting for topics considered most important for systematic review. Five priority systematic reviews were then carried out by the researchers.	Young people (*n* = 13 for meeting 1, and *n* = 13 for meeting 2), from an existing young people’s public involvement in research group (the existing group had 25 members, who were recruited via advertisements. The group met monthly).	Face-to-face meetings Meetings were 1 h. Meetings involved short presentations and group discussion. Meetings were ‘supplemented with an online discussion forum’. Meeting 1 and online discussion forum generated list of health topics. Meeting 2 agreed a final list and voted individually to prioritise.	Consensus decision-making techniques: voting/ranking	No information provided	Members were not rewarded directly from researchers. However, they received £15 vouchers for their monthly participation (not specific to this research project), had food and transport provided and were eligible for an annual residential teambuilding activity.
Liabo [46]	A participatory approach was used to involve a group of young people in all stages of a review.	Young people (total of 20 people across all meetings; 5 people only came to 1 meeting, others to multiple meetings; 2 came to all meetings). Target group was young people (up to age of 25) with experience of being in care.	20 review meetings	‘Participatory methods’	The study was granted ethical approval by the Institute of Education ethics committee (application number FCH 62) in November 2007.	This study adapted the payment regulations operated by PAS, which was a fixed amount per hour, specified for meetings, training (half of meetings) and presentations (higher than meetings).
Liu et al. [47]	User engagement was undertaken throughout our project through the inclusion of lay members on our Independent Project Steering Committee; in addition, we held two user conferences, one to launch the study and one to share and discuss our preliminary findings.	A total of 81 delegates attended the first conference, and 71 the second conference, from a wide variety of stakeholder organisations.	Independent Project Steering Committee Two meetings: 1. To obtain feedback on the research proposal and methods 2. To present and discuss preliminary findings	–	Ethical arrangements outlined in protocol, but unclear if formal ethical approval applied for or granted	No information provided
Martin et al. [36]	A PPI (patient and public involvement) event was organised to facilitate members of the public in the East of England to talk about their views on population screening for dementia. The aim was to contextualise the findings of a systematic review for a British audience.	Purposive approach (50 invited, 36 attended)	Meeting (1 all day public event). Held during the final stages of the systematic review	‘Quasi-focus group format’	No information provided	In recognition of participants’ time and to mitigate the risk of participant dropout, a fixed monetary honorarium of £80 was offered and reminder calls were made in advance of the event. Costs for supportive care were provided to partners if they attended without their care recipients, and travel costs
McConachie et al. [48]	A ‘multifaceted approach to consultation’, including (1) consultation with young people with ASD, in groups and by email; (2) survey of health professionals; (3) consultation with parents—3 meetings throughout review process; (4) multiple stakeholder discussion day about the preliminary conclusions of the review.	People with ASD—12 young people and 8 adults responded. Health professionals—838 survey respondents Parents—7 participated in one or more meetings Multiple stakeholder day—16 people	Methods included meetings, survey and emails.	Q-sort for rating agreement with outcomes	No information provided	Young people: ‘Each respondent was given a shopping voucher in acknowledgement of their contribution’. Parents: ‘Parents were given a financial acknowledgement in addition to travel expenses, to recognise their time and expertise at each attendance’.
McCusker et al. [49]	A literature review on collaborative mental health care for depression was completed and used to guide discussion at an interactive workshop. The workshop was held as part of pre-conference activities at the June 2011 Canadian Conference on Collaborative Mental Health Care in Halifax, Nova Scotia.	We invited a spectrum of stakeholders to participate, aiming to have roughly equal representation of 4 groups: primary care providers, mental health providers, decision makers and consumers.	Workshop (*n* = 40; 9 members of planning committee and 31 invited stakeholders) Survey, to rate agreement with themes arising from workshop (*n* = 21/42, 50% response rate)	Thematic analysis of workshop discussion	The protocol was approved by the St Mary’s Hospital Research Ethics Committee. Participants provided oral but not written consent to participate. Permission was requested at the workshop for the proceedings to be audio-recorded and photographed, and for the participants’ names and affiliations to be listed in the final report. As one person did not agree to audio recordings, no recordings were made.	No information provided
McGinn et al. [56]	A Delphi study among Canadian representatives of actual or potential EHR users to confirm the findings of the systematic review and to prioritise the key barriers and facilitating factors for EHR implementation in Canada.	106 participants: 14 physicians, 30 healthcare professionals, 33 managers, 29 health information professionals (0 patient representatives due to low recruitment).	Delphi—3 rounds: 83 participants completed round 1, 69 round 2 and 63 round 3.	Delphi approach	Ethics approval for the study protocol was received from the Research Ethics Board of the Centre Hospitalier Universitaire de Québec (approved January 23, 2009; ethics number 5-08-12-06).	No information provided
Morgan et al. [58]	As part of a partnership approach for a wider project relating to incentive mechanisms for smoking cessation in pregnancy and breastfeeding, 2 mother-and-baby groups were recruited and were co-applicants on the wider project.	Members of 2 existing groups (groups of around 12 people). Groups were mother and baby/toddler groups.	15 meetings (during project, not just systematic review component). Members contributed to interpretation of systematic review findings by providing feedback on a number of vignettes of studies included in the evidence synthesis.	‘Participatory’	Although the service user collaborators were independent or local government representatives, rather than NHS groups, we considered it preferable to gain ethics committee approval before active engagement, particularly because the groups had not been involved in research before.	No information provided
Oliver et al. [37] (‘correlational’ review)	A one-off workshop/meeting with members of an existing group. The workshop was scripted. Ground rules were established. Literature had been screened but not synthesised: involvement explored variables and pathways that had been examined in the literature, aiming to contribute to a theoretical framework to help synthesise study findings.	Young people, aged 12–17 years (approximately 12)	One meeting (2.5 h). Format: discussion, ranking of themes	Used methods for ranking of importance (stickers and ranking cards), but these did not directly inform decision-making	No information provided	No information provided
Oliver et al. [37] (‘views’ review)	A one-off workshop/meeting with members of an existing group. The workshop was scripted. Ground rules were established. The synthesis of literature was almost complete; the aim of involvement was to provide a check on the credibility of the synthesis and develop review implications.	Young people, aged 12–17 years (approximately 12)	One meeting (2.5 h). Format: discussion, ranking of themes	As above	No information provided	No information provided
Oosterkamp et al. [54]	RAND-e modified Delphi (2 rounds) Group (consensus) meeting The purpose of the involvement was to develop consensus on statements on the prevention of WSL during orthodontic treatment with fixed appliances.	The expert panel comprised 11 representatives, all graduated dentists, from research, education, orthodontics, cariology, general dentistry and advisory general dental practitioners from health insurance companies.	RAND-e modified Delphi procedure (involves considering patient vignettes). Consensus meeting	Delphi approach	No information provided	No information provided
Pearson et al. [50]	Two meetings at different stages in the review process. Meeting 1 took place at the start of the review and aimed to ‘sharpen the focus of the review so that it would be relevant to those directly involved in the implementation’. Meeting 2 took place when a draft of the review was complete, aimed at getting feedback on this draft.	Professionals (*n* = 10)—primary and secondary school-level educational professionals and senior academics linked to the review	Two face-to-face meetings (length unclear)	–	No information provided	No information provided
Pollock et al. [38]	Three meetings at different stages in the review process. Meeting 1 aimed to explore how the intervention could be categorised within the review. Meeting 2 aimed to explore and agree on specific strategies to update and amend the current Cochrane systematic review, based on the decisions made during meeting 1. Meeting 3 aimed to explore the perceived clinical implications of the findings.	Patients, carers, professionals (*n* = 13)	Three face-to-face meetings (half day meetings) Additional contact by email throughout review.	Nominal group technique	The project was approved by the Glasgow Caledonian University School of Health and Life Sciences ethics committee (Reference: HLS12/40).	We costed for the direct expenses associated with involvement (that is, travel, subsistence), but not for any funding to pay for the time of group members.
Rees et al. [51]	An advisory group, which met 3 times during the project. The specific tasks for the group included: • Advising on the most appropriate terminology relevant to the subject area; • Identifying the literature, particularly unpublished reports; • Identifying and prioritising a priori outcomes for analysis; • Informing decisions the review team had to make at key stages of the review; • Helping to disseminate the work through incorporating its findings into members’ respective areas of work, and publicising the review to colleagues and associates.	Researchers/academics, policy specialists, voluntary sector workers, and a practitioner, representing a number of organisations (*n* = 7)	Three face-to-face meetings, of around 2 h. Format—meetings were ‘semi-informal’, involving presentations, discussions and explicit consensus methods (voting)	Consensus decision-making techniques: voting	No information provided	No information provided
Saan et al. [55] (review 1)	‘A partnership between the commissioner and researchers’	Academic experts (*n* = 2)	‘discussion’ Format unclear	–	No information provided	No information provided
Saan et al. [55] (review 2)	Participants in an anonymous online Delphi process: ‘39 experts from the Netherlands (34 researchers and five policy advisors) were invited by email. They were contacted through the contact list of a recent expert meeting on self-management in the Netherlands and through the professional network of members of the research group.’	Academic experts (29 invited: 20 completed first round of Delphi, 17 second round, 16 third round)	Anonymous online Delphi	Consensus decision-making techniques: Delphi	No information provided	No information provided
Serrano-Anguilar et al. [39]	Three rounds of electronic Delphi. Round 1—an open questionnaire to explore the treatments used by patients and perceived health problems associated with their disease. Round 2—aimed at prioritising the health problems identified in the first round. Round 3—aimed at reaching final consensus.	Patients with DA (*n* = 53)	Electronic Delphi (3 rounds)	Consensus decision-making techniques: Delphi method	This study was approved by the Ethics Committee of the Canary Islands since its coordination was carried out by the Planning and Evaluation Unit of the Canary Islands Health Service	No information provided
Smith et al. [52]	A ‘reference’ group was set up for the project. There were 3 face-to-face meetings at different stages in the review. Meeting 1 was aimed at sharing ideas and agreeing how the group would work. Meeting 2 discussed the scope and remit of the review. Meeting 3 was aimed at identifying key messages from the findings and planning dissemination. Additional communication between researchers and the reference group occurred by telephone conversations, email, project website and newsletters.	Service user and carer advocacy group members (*n* = 26)	Three face-to-face meetings (length unclear)	–	No information provided	No information provided
Steward and Oliver [40]	The wider project aim was to ensure that policies and resources arising from the systematic review were relevant to parents, taking into account their experiences and views. Policy and resources were developed by 12 multi-disciplinary expert groups. Parents were included in these expert groups to share experiences and join discussions.	22 parents, with experiences of newborn screening	Group meetings (3), over an 18-month period.	–	No information provided	Paid: • Parents’ travel expenses to and from meetings • For coffee at pre-meetings • Honoraria for parents equivalent to £17 per hour at each meeting, given in gift vouchers: this came to £75–£100 for each meeting • A fee to organisations that assisted with recruitment, in recognition of the support these organisations provided to parents. The fee was either £8 per hour of meeting attended by their members, or later, because this became too difficult to administer, a one-off £50 fee per parent recruited.
Vale et al. [41]	Patient research partners had an initial face-to-face meeting, then contributed to a number of review activities, including ‘providing feedback on the detailed information folders, helping to trace contact details for trial investigators, learning about data management and analysis and contributing to regular project newsletter’	(1) Reference group—professionals, patient/charity representatives, patient (*n* = 7) (2) Patient research partners—women who had received treatment for cervical cancer (*n* = 6)	‘Patient research partners’—also contributing to another research project—around 6 meetings/year. Additional communication via email	–	No information provided	No information provided

Note that within this table there are 32 reviews described, as 2 of the 30 included papers describe 2 reviews, and data has been extracted separately for these at this stage (Oliver et al. [37] ‘correlational’ review and ‘views’ review; Saan et al. [55] review 1 and review 2)

#### Review aim/focus

Table 5 states the aim and focus of the 32 systematic review. Sixty-eight percent were focussed on one of the ICD-10 categories; with mental and behavioural disorders being the most common health topic (22%). Sixteen percent were focussed on a specific intervention rather than a disease area, most commonly on a public health intervention (12%). The remaining 16% were focussed on either research or another topic.

A majority of the reviews (56%) synthesised both qualitative and quantitative evidence, while 19% only included quantitative studies and 12.5% only included qualitative studies. The type of evidence included was unclear for 12.5%. Two of the reviews described using a ‘realist’ review methodology, and 2 were Cochrane reviews of randomised controlled trials.

#### People involved

Seventy-eight percent of the systematic reviews involved patients, carers or family members, while in one (3%), the people involved were peer support workers. In 19% of systematic reviews, the only people involved were professionals or academic experts, although one of these [56] aimed to recruit patient representatives, but failed to do so. Where there were face-to-face meetings, the number of stakeholders involved ranged from 2 to 27; where there were one-off events, often advertised as open to the general public, the numbers of stakeholders involved ranged from 15 to 81; where involvement did not require a face-to-face meeting, for example using an electronic Delphi or survey, the numbers invited ranged from 29 to 340 (see Table 6).

#### Geographical location (from which stakeholders were recruited)

The majority of the involvement occurred in the UK, with two thirds (66%) of papers describing UK-based activities. Of the remaining 34%, 2 recruited people from across Europe, 3 were carried out in Canada, 3 in the Netherlands and 1 in Australia, USA and Spain.

#### How people were recruited

For 69% (22/32) of the systematic reviews, people were personally invited to be involved. This involved personal invitations to known people (in 12/22; [29, 31, 39, 41–44, 48, 54, 55, 57]); personal invitations to an existing group or groups (in 6/22; [34, 35, 37, 46, 58]); or purposive sampling, using similar methods as sampling for qualitative research (in 4/22; [32, 38, 40, 56]). For a further 7/32 of the systematic review, involvement opportunities were advertised to the general population, often snowballing information out via target groups and organisations, and anyone who volunteered could get involved [25, 36, 47, 49, 50, 52, 53]. A combination of different recruitment strategies was used for 1 systematic review [33], and the method of recruitment was unclear for 3 systematic reviews [30, 45, 51].

#### Format of involvement

The format of involvement comprised direct, face-to-face interaction in 81% and an electronic Delphi method or survey in 19% of the systematic reviews. The face-to-face interaction was either in the format of a meeting (53%; [29, 31, 32, 34, 35, 38, 40–44, 46, 50, 52, 55, 58]), a larger workshop or public event (19%; [25, 36, 37, 47, 49]) or a combination of both of these (9%; [33, 45, 48]). In each of the 6/32 systematic reviews which used an electronic Delphi method, there was a specific and focussed aim of stakeholder involvement; in 4/32 [30, 53, 54, 56], this was broadly related to reaching consensus on factors, recommendations or statements arising from the results of the systematic review, and in 2/32 [39, 55], this was to reach consensus on the topic or focus of the systematic review.

#### Amount of involvement

Where there was direct face-to-face interaction, there could be between 1 and 20 meetings or events. The majority (83%) of the 24 reviews providing this information held 4 or less meetings (median 2 meetings), while one held 5 meetings plus 3 public workshops [45]. Three held multiple meetings (12, 15 and 20 respectively by [25, 46, 58]); in each of these three examples, the approach is described as ‘participatory’. Where reported, the length of face-to-face meetings varied from 1 h to ½ day. Generally, the Delphi approach involved three rounds of an electronic survey, although in one example after two rounds of Delphi voting there was a direct face-to-face consensus meeting [54].

#### Ethical approval

Details of ethical approval were reported for 31% of systematic reviews; for details, see Table 6. One paper reported that ethical approval was sought but not required [44]. No details relating to ethical approval were provided by the remaining 66% of papers.

#### Financial compensation

Expenses (such as travel, accommodation and care costs for family members) were reported to be paid to people involved in 25% of systematic reviews; in two, this was expenses only, while in six money or a voucher was provided in addition to expenses (see Table 6). No details relating to financial compensation are reported in the remaining 75% of systematic reviews.

#### Tools or method of reporting involvement

Thirty-four percent of the included papers had a clear method of reporting involvement. Four used some sort of tool, framework or checklist: Concannon et al. [42] developed and used a 7-item question for reporting stakeholder involvement in research, Liabo [46] used a framework for considering impact of involvement, Martin et al. [36] reported an evaluation based on reporting standards within Guidance for Reporting Involvement of Patients and the Public (GRIPP) checklist [59] and Saan et al. [55] developed and used a Tool for Recording and Accounting for Stakeholder Involvement (TRASI). Seven specifically reported activities which groups of stakeholders were involved in within a specific section of text, tables or supplementary files [30, 35, 37, 38, 47, 50]. Within the remaining 66% of systematic reviews, information about methods of involvement was not reported within a particular section, table or file, but was distributed throughout the paper.

#### Evaluation of the methods of involvement

None of the 32 studies carried out any formal evaluation of the impact of involving stakeholders; however, 28% collected data relating to the views and experiences of people involved. Of these, four used a questionnaire to elicit the views and experiences of stakeholders [29, 30, 36, 41]; three held a discussion with stakeholders in which they were encouraged to share or reflect on their experiences and perspectives [31, 33, 47]; and two had both a questionnaire and a discussion [38, 44]. In addition, Liabo [46] reported data arising from audio recordings and minutes of all meetings, Hyde et al. [34] described ‘impact’ within a table, while the reflections of the researchers on the process of involvement were discussed by others [35, 37, 40, 52].

## Discussion

### Key findings: evidence-base relating to stakeholder involvement in systematic reviews

We identified 291 papers describing stakeholder involvement in systematic reviews. Approximately two thirds of published examples describe UK activities, but we found examples from at least 24 countries. Reporting of who was involved, in what ways and at what stage in the review process was generally very poor, and the majority of the papers (59%) were judged to provide few details and/or an inadequate description of the method or approach of involving stakeholders. Thirty percent of systematic review teams clearly involved patients/carers, but in many cases (41%), the stakeholders involved health professionals, academic experts or representatives of patient organisations, but not patients or their family members.

We identified 30 papers, describing 32 systematic reviews, which we judged to have sufficiently comprehensive reporting to allow a more in-depth synthesis of methods or approaches to the involvement of stakeholders in systematic review. We have described key features of how stakeholders have been involved in systematic reviews, using data from these 32 examples. However, it was notable that, despite the selection of systematic reviews which were judged to provide a comprehensive description of one or more method of involvement, there was still inadequate (or absence of) reporting of a number of features in which we were interested. For example, the majority of papers did not provide any information relating to ethical approval or financial compensation to the stakeholders involved. A key contributing factor to the poor reporting relating to aspects of how stakeholders were involved may have been the lack of a tool or standardised method for reporting. On the few occasions where a particular tool has been used to support reporting of information relating to involvement, the tool has often been developed specifically by the systematic review authors. In many cases, the method of reporting comprises a written description of the activities in which stakeholders have been involved, but we found inconsistencies in the type of information presented and the location of this information within published papers.

### Implications: methods of involving stakeholders in systematic reviews

The evidence which we have synthesised demonstrates that actively involving stakeholders within systematic reviews is feasible, and can be incorporated into a wide range of different types of systematic review. While there can be considerable variation in how stakeholders are involved, and the types of stakeholders who are involved, and there is currently an absence of evidence to directly inform choices for methods of stakeholder involvement within future reviews, a number of implications can be drawn from our synthesised evidence. In particular, evidence drawn from the 32 examples explored in this review can highlight some of the methodological decisions which may be made when planning stakeholder involvement in future reviews. These include:

- Will people directly affected by the healthcare topic addressed within the systematic review (i.e. individual patients, carers or family members) be involved? Will health professionals, academic experts or representatives from patient organisations be involved?
- How to find people to involve? Within our 32 examples, we found two key methods of recruiting stakeholders to be in systematic reviews; in the majority of our examples, there were personal invitations to known individuals or groups, but in some cases, recruitment occurred through advertising to the general population in order to get stakeholders to volunteer to be involved.
- How will people be involved? Within our 32 examples, two distinct methods of involving people in a systematic review were identified: (i) face-to-face meetings or events or (ii) electronic Delphi method. Where there were face-to-face meetings, these could be attended by invited participants only or could be an open event or workshop to which members of the public are invited to attend. Invited participants may only attend a small number (often between 1 and 4) of meetings during the course of a systematic review, but this may be much more where a participatory approach is used.
- How many stakeholders to involve? The current evidence base indicates that the number of stakeholders depends on the way in which they will be involved. Evidence from the 291 papers in our synthesis shows that 1 stakeholder may be a co-author on a systematic review, 2–10 stakeholders may be members of a steering group, 5–50 stakeholders may attend face-to-face meetings or focus groups and 20–400 stakeholders may participate in Delphi rounds or attend events or conferences.
- Use of research methods? Our examples highlighted that the following research methods have sometimes been incorporated into stakeholder involvement in systematic reviews: focus groups, interviews and a number of consensus decision-making techniques such as Delphi, Nominal Group Technique and voting/ranking processes.

Other issues to consider when planning stakeholder involvement in systematic reviews are whether ethical approval will be required and resources for payment of expenses and any other financial compensation or reward.

Although there is insufficient evidence to directly inform choices relating to who to involve and in what way, the findings arising from the 32 papers identified in this review have been used to produce, in collaboration with Cochrane Training, freely available online learning material and resources [60]. There have been many urgent calls for high-quality training materials, reporting guidelines and examples of best practice to support active stakeholder involvement and to enhance the relevance, usefulness and accessibility of systematic reviews [2, 16, 18, 33, 61]; the evidence from this review therefore can arguably currently play a key role in learning and support relating to active stakeholder involvement in systematic reviews.

### Implications: reporting stakeholder involvement in systematic reviews

Recording and reporting of stakeholder involvement is important, both to ensure transparency in relation to the contributions and roles of different stakeholders within the review process and to contribute to the evidence base relating to this field. This scoping review highlights that the current reporting of involvement in systematic reviews is very poor and sometimes absent, and rarely provides a comprehensive description of who was involved and in what way. While there are a number of tools and frameworks which review authors could consider using (e.g. [36, 46, 55]), there is not currently any tool, guidance or recommendations specifically designed to support reporting of involvement within systematic reviews. Generic guidance relating to the reporting of stakeholder involvement in research has recently been updated (GRIPP2, [62]); however, this guidance has not been specifically tested for use with systematic reviews and has lacked international input during development. It is clear that there is an urgent need for improved reporting of involvement of stakeholders in systematic reviews. Such reporting should enhance the ability to develop evidence-based guidance around how to involve stakeholders in systematic reviews, and to explore and evaluate the impact of involvement.

### Limitations

#### Identification of relevant systematic reviews and data extraction

It is unlikely we identified all relevant examples of stakeholder involvement in systematic reviews, as we adopted a pragmatic search approach aimed at efficiency within project time and resource constraints. This was compounded by poor reporting and inconsistent terminology in this area. We believe it is highly likely that there are many systematic reviews where stakeholders played a key role that our methods could not identify. Our decision to exclude titles without abstracts and review protocols at the study selection stage may have introduced publication bias into our results, with a bias toward inclusion of papers published in peer-reviewed journals. Only one review author extracted data from the included studies, and there is the potential that this may have introduced bias and errors in extraction. In an attempt to improve transparency and reduce data extraction errors, we copied and pasted data verbatim from included papers into an electronic data extraction sheet. This is reported in the table of included studies (Additional file 7).

#### Judgement to identify those with most comprehensive description

The agreement between independent reviewers when applying the ‘comprehensiveness’ judgement to a subset of papers indicated that there were disagreements on around 17% of ‘green’ categorisations. We did not have the time or resources to have independent judgement on a higher proportion of studies. We are therefore not confident that our subset includes all papers which may provide an adequate description of some parts of the methods of involving people in a review. However, as the aim of this phase was to identify and describe methods of involvement from examples of systematic reviews, the impact of potentially falsely including or excluding a paper from this subset was perceived to be low. We present the included ‘green’ papers as examples of systematic reviews in which there was involvement of stakeholders and take care to stress that these are examples rather than a comprehensive sample.

Our judgement of the comprehensiveness of the description of the methods was not a judgement of the quality of the involvement methods and only relates to the depth of the description of stakeholder involvement provided in the identified paper. Over half (54%) of the 291 included papers were reports of a guideline or recommendation, but only 2 of these were judged as ‘green’ for comprehensiveness of description. A potential explanation for this finding could be that stakeholder involvement is generally a core component of guideline development, but the primary focus of related journal publications is often the key clinical messages and implications, rather than the methods of the guideline, which are often fully described elsewhere. A judgement of ‘amber’ or ‘red’ for the comprehensiveness of the description of the method of involvement in the published paper is not an indication either that the quality of the methods was poor or that details of methods of stakeholder involvement are not available elsewhere.

## Conclusion

This systematic review summarises evidence relating to the involvement of stakeholders in systematic reviews. We identified a relatively large number (291) of papers reporting stakeholder involvement in systematic reviews, but the quality of reporting was generally very poor. The level of reporting of involvement of stakeholders in systematic reviews, and the inconsistencies in which this is reported, must be improved so that guidance around how people can be involved in systematic reviews can be developed and the impact of involvement explored. This scoping review lends support to calls for high-quality training materials and examples of best practice to support active patient and public involvement and enhance the relevance, usefulness and accessibility of systematic reviews [2, 16, 18, 61, 63]. We identified a subset of 30 papers which we judged to provide a comprehensive description of stakeholder involvement in systematic reviews, and used these examples to summarise different ways in which stakeholders have been involved in systematic reviews. These examples arguably currently provide the best available information to inform and guide decisions around the planning of stakeholder involvement within future systematic reviews. This evidence has been used by Cochrane Training to develop online learning resources relating to how to involve people in systematic reviews [60], and has been used to develop a framework for describing stakeholder involvement in systematic reviews (Pollock A, Campbell P, Struthers C, Synnot A, Nunn J, Hill S, Goodare H, Morris J, Watts C, Morley R: Development and application of a framework to describe how stakeholders have been involved in systematic reviews, submitted).

## Additional files

Additional file 1: Protocol deviations. (DOCX 31 kb)

Additional file 2: Search strategy. (DOCX 23 kb)

Additional file 3: Year of publication of included studies. (DOCX 25 kb)

Additional file 4: Data extraction (scoping review). (DOCX 35 kb)

Additional file 5: Agreement between independent reviewers for judgements. (DOCX 32 kb)

Additional file 6: Table of excluded studies. (DOCX 50 kb)

Additional file 7: Table of included studies. (XLSX 166 kb)

## Abbreviations

ACTIVE: Authors and Consumers Together Impacting on eVidencE
AMED: Allied and Complementary Medicine Database
CDSR: Cochrane Database of Systematic Reviews
CENTRAL: Cochrane Central Register of Controlled Trials
CINAHL: Cumulative Index to Nursing and Allied Health Literature
DARE: Database of Abstracts of Reviews of Effectiveness
DoPHER: Database of Promoting Health Effectiveness Reviews
EBSCO: An online platform for accessing research databases
Embase: Excerpta Medica Database
EPPI-centre: Evidence for Policy and Practice Information and Co-ordinating Centre
GRIPP: Guidance for Reporting Involvement of Patients and the Public
HTA: Health Technology Assessment
ICD-10: International Statistical Classification of Diseases and Related Health Problems 10th Revision
MEDLINE: On-line Medical Library database
OVID: An online platform for accessing research databases
PDQ-Evidence: (‘Pretty darn quick’) database of systematic reviews of health systems evidence
