# Supplementary material for: Stakeholder involvement in systematic reviews: a scoping review
Source: Syst Rev. 2018 Nov 24;7:208. doi: 10.1186/s13643-018-0852-0 (PMC6260873; doi:10.1186/s13643-018-0852-0)
Supplement: Supplementary file 1 — Protocol deviations. (DOCX 31 kb) [file 13643_2018_852_MOESM1_ESM.docx]

**Additional File 1: Protocol deviations**

Published protocol: Pollock A, Campbell P, Struthers C, Synnot A, Nunn J, Hill S, Goodare H, Watts C, Morley R: Stakeholder involvement in systematic reviews: a protocol for a systematic review of methods, outcomes and effects. Research Involvement and Engagement 2017, 3:9

| **Deviation** | **Explanation / justification** |
| --- | --- |
| Protocol described a scoping review plus two contingent syntheses. Here we present the scoping review plus one of the planned contingent syntheses (described as “Synthesis A” in published protocol). The synthesis of studies of effectiveness (“Synthesis B” in published protocol) is not reported here. | The volume of information that synthesised within the scoping review plus synthesis A was considerable, and it was not considered possible to incorporate further syntheses into the one paper.  (In fact we identified no studies of effectiveness, which would form Synthesis B, but many other papers do discuss the issue of effectiveness and therefore we feel that it would be appropriate to consider this in greater depth in a separate paper). |
| Protocol stated that we would contact authors for missing information. | Due to the higher than anticipated number of included studies and resource constraints this was not done. We considered that we had sufficient information from the available studies. |
| Protocol stated that we would extract additional data from papers judged as ‘amber’, as well as from those judged as ‘green’. We only extracted the additional data from those judged as ‘green’. | The number of papers judged as ‘green’ was far higher than anticipated, and we therefore considered that we had sufficient information from the ‘green’ studies, and insufficient resource to explore the ‘amber’ studies as well. |
| Planned searches of Epistemonikos and PDQ-Evidence were not completed | This was due to difficulties implementing a suitably sensitive search. |
| We made the post-hoc decision that we would also exclude reviews that described contact with experts as part of the search strategy. | We had not considered this issue within the protocol, and therefore had to clarify this. Contacting experts as part of the search strategy is a common process in a systematic review and not generally considered ‘involvement’. We therefore clarified this point and excluded studies if this was the only ‘involvement’ reported. |
| We made the post-hoc decision to exclude evidence relating to organisations. | Although we had stated in the protocol that we would include evidence relating to organisations, we revisited this during team discussions. Evidence relating to involvement at the level of organisations had previously been reviewed (Morley 2016), and our initial exploration of these papers highlighted that the evidence was not aligned with the data which we sought to extract (I.e. relating to methods of involving people in individual reviews). |
| We made the post-hoc decision to categorise evidence relating to the focus of the systematic review. This decision occurred after extraction of text / quote which described the focus of each systematic review. | Categorised according to focus on a specific disease or health area (based on ICD-10 headings) or, if the focus was not on a specific disease or health area, categorised according to type of intervention (based on ICHI headings) or as focussed on research methods, or ‘other’.  (see operationalisation of data extraction for details of ICD-10 and ICHI headings). |
| We made the post-hoc decision to categorise the type of evidence synthesised within each systematic review. This decision occurred after extraction of text / quote which described each systematic review. | Categories of qualitative / quantitative / mixed or unclear were applied. (see operationalisation of data extraction for details of ICD-10 and ICHI headings). |

Morley RF, Norman G, Golder S, Griffith P: **A systematic scoping review of the evidence for consumer involvement in organisations undertaking systematic reviews: focus on Cochrane.** *Research Involvement and Engagement* 2016, **2:**36.
