# Supplementary material for: Stakeholder involvement in systematic reviews: a scoping review
Source: Syst Rev. 2018 Nov 24;7:208. doi: 10.1186/s13643-018-0852-0 (PMC6260873; doi:10.1186/s13643-018-0852-0)
Supplement: Supplementary file 3 — Year of publication of included studies. (DOCX 25 kb) [file 13643_2018_852_MOESM3_ESM.docx]

**Additional File 3: Year of publication of included studies** (and judgement of comprehensiveness)

Judgement of comprehensiveness of included studies:

- ‘Green’ - Comprehensive description of one (or more) specific method or approach to the involvement in systematic reviews. Description sufficient to enable replication of methods.
- ‘Amber’ - A brief or partial description of one (or more) specific method or approach to the involvement in systematic reviews. Description sufficient to enable partial replication of methods.
- ‘Red’ - Few details provided and/or inadequate description of the method or approach of involvement. Description insufficient to enable any replication of methods.
