# Supplementary material for: Stakeholder involvement in systematic reviews: a scoping review
Source: Syst Rev. 2018 Nov 24;7:208. doi: 10.1186/s13643-018-0852-0 (PMC6260873; doi:10.1186/s13643-018-0852-0)
Supplement: Supplementary file 4 — Data extraction (scoping review). (DOCX 35 kb) [file 13643_2018_852_MOESM4_ESM.docx]

**Additional File 4 – Data extraction (scoping review)**

Operationalisation of data extraction items

| Data extraction item | Operationalisation |
| --- | --- |
| Bibliographic information | Full citation |
| **Study author** | First author surname |
| **year of publication** | Year of publication |
| type of paper | Select:   - Systematic review - Report of guideline / recommendation - Description of methods of involvement - Other |
| **stated aim** | **Relevant quote from paper** |
| Topic / focus of systematic review | Categorised according to focus on a specific disease or health area (based on ICD-10 headings*) or, if the focus was not on a specific disease or health area, categorised according to type of intervention (based on ICHI headings**) or as focussed on research methods, or ‘other’. |
| **Methodological focus / study methodology** | Relevant quote from paper (e.g. methods section from abstract) |
| **Type of evidence synthesised** | Select:   - Qualitative evidence - Quantitative evidence - Mixed (i.e. qualitative and quantitative) - Unclear |
| **Description of reported method(s) or approach(es) to involvement of members of public** | Relevant quote or extracts from paper, giving overview of methods of involving people |
| **Details/experience of people involved (patients, carers, professionals, policy makers etc)** | Relevant quote or extract from paper, describing who was involved (background, numbers etc) |
| **were patients or carers involved?** | Select:   - Yes - No - Unclear |
| Country | Country of people involved or, if this was not stated, country of lead author (from contact details) |
| **at what stage were people involved?** | Select:   - setting scope / review questions - interpreting results after review completion - 1 and 2 - throughout/within the review process - unclear |
| **Judgement of how comprehensive the description of methods of involvement is** | Select:   - GREEN = comprehensive description of one (or more) method or approach to involvement. Description sufficient to enable replication. - AMBER = brief or partial description of one (or more) method or approach to involvement. Description sufficient to enable partial replication. - RED = few details provided and/or inadequate description of the method or approach. Description insufficient to enable replication - Not applicable = paper does not describe involvement in a systematic review - Unclear / uncertain |
| **If not applicable, state reason why** | State reason |

*International Statistical Classification of Diseases and Related Health Problems 10th Revision(ICD-10) headings (http://apps.who.int/classifications/icd10/browse/2010/en):

| I Certain infectious and parasitic diseases |
| --- |
| II Neoplasms |
| III Diseases of the blood and blood-forming organs and certain disorders involving the immune mechanism |
| IV Endocrine, nutritional and metabolic diseases |
| V Mental and behavioural disorders |
| VI Diseases of the nervous system |
| VII Diseases of the eye and adnexa |
| VIII Diseases of the ear and mastoid process |
| IX Diseases of the circulatory system |
| X Diseases of the respiratory system |
| XI Diseases of the digestive system |
| XII Diseases of the skin and subcutaneous tissue |
| XIII Diseases of the musculoskeletal system and connective tissue |
| XIV Diseases of the genitourinary system |
| XV Pregnancy, childbirth and the puerperium |
| XVI Certain conditions originating in the perinatal period |
| XVII Congenital malformations, deformations and chromosomal abnormalities |
| XVIII Symptoms, signs and abnormal clinical and laboratory findings, not elsewhere classified |
| XIX Injury, poisoning and certain other consequences of external causes |
| XX External causes of morbidity and mortality |
| XXI Factors influencing health status and contact with health services |

**Derived from International Classification of Health Interventions (ICHI) headings (<http://www.who.int/classifications/ichi/en/>, [www.mdpi.com/1660-4601/15/1/145/pdf](http://www.mdpi.com/1660-4601/15/1/145/pdf) ):

| ICHI Medical & surgical intervention |
| --- |
| ICHI Interventions to support functioning |
| ICHI Public health interventions |
