# Supplementary material for: Stakeholder involvement in systematic reviews: a scoping review
Source: Syst Rev. 2018 Nov 24;7:208. doi: 10.1186/s13643-018-0852-0 (PMC6260873; doi:10.1186/s13643-018-0852-0)
Supplement: Supplementary file 5 — Agreement between independent reviewers for judgements. (DOCX 32 kb) [file 13643_2018_852_MOESM5_ESM.docx]

**Additional File 5:** Agreement between independent reviewers in relation to the judgement of comprehensiveness of description

One reviewer (AP) judged the comprehensiveness of description of all included studies. An independent reviewer (CS) judged the comprehensive of description of a random selection of 42 of these studies. The table below summarises the agreement (and where there was disagreement) between reviewers:

| **Agreement?** | **Percentage of studies (number)** | **Notes** |
| --- | --- | --- |
| Exact agreement (i.e. both reviewers made same judgement) | 57.1% (24) | - |
| Partial agreement, of no consequence to review results (i.e. one judgement of ‘red’ and one of ‘amber’) | 7.1% (3) | These differences in judgement will have had no consequence on the systematic review results, as papers judged to be “amber” and “red” were dealt with in the same manner. |
| Partial agreement of minimal consequence to review results (i.e. one judgement of ‘red’ and one of ‘not applicable’, or one of ‘amber’ and one of ‘not applicable) | 19.0% (8) | These differences in judgement will have had some consequence on the systematic review results.  However:   - in 6/8 occurrences, reviewer AP applied a judgement of Amber/Red whilst reviewer CS applied a judgement of Not Applicable. - In 1/8 occurrences one reviewer had mistakenly included a protocol, when this should have been judged to be “not applicable” - In 1 /8 occurrences reviewer AP judged a paper to be ‘not applicable’ whilst CS judged ‘Red’   The more inclusive tendency of reviewer AP suggests that there is a risk that around 14% (6/42) of the red/amber papers may be falsely included; while there was a low chance (2.3%, 1/42) red/amber papers have been falsely excluded). |
| Partial agreement with potential impact to synthesis (i.e. one judgement of ‘amber’ and one of ‘green’) | 14.3% (6) | Reviewer CS applied a judgement of ‘green’ in 6 papers, when reviewer AP applied a judgement of ‘amber’.  Exploration of the content of these 6 papers found that 4 were reports of guidelines using a Delphi approach; one was an HTA review which had incorporated stakeholder interviews; one was a realist review in which data had been generated from an expert panel.  Discussion with a 3^rd^ reviewer (PC) reached consensus that the description was not fully comprehensive, and inclusion of these papers as ‘green’ would not change the conclusions of the synthesis. |
| Disagreement (i.e. one judgement of ‘green’ and one of ‘not applicable’) | 2.4% (1) | For 1 paper (Martin 2015), AP applied a judgement of ‘Green’ and CS a judgement of ‘not applicable’. CS’s justification for this judgement related to the stage of involvement (the systematic review was completed prior to any stakeholder involvement).  Discussion with a 3^rd^ reviewer (PC) reached consensus that as the stage had been transparently reported within the synthesis of ‘green’ studies, the inclusion of this paper did add a further valuable perspective on different approaches and types of involvement. |

On discussion it was agreed that no changes should be made to the judgements applied by AP. However the following key points should be noted:

- 64.2% (57.1% + 7.1%) of judgements are at low risk of bias and independent judgement would not change the results
- There is a moderate risk (around 14%) of over inclusion of papers, with judgements of red or amber applied to papers which should possibly be excluded. In contrast we found a low risk of over exclusion of papers (2.3%)
- There is a moderate risk that some papers (14.3%) may be categorised as amber when the description may be sufficient for a green categorisation. However the inclusion of these papers as ‘green’ is unlikely to bring any new findings which would change the conclusions of the synthesis.
- There is a small risk that some papers are included as “green” when the paper did not describe involvement in a systematic review (but rather involvement after a systematic review was completed). This occurred with one paper within our random sample, but this has been left as ‘green’ as it was agreed that the stage of involvement was transparently reported and that the inclusion of this paper did add a further valuable perspective on different approaches and types of involvement.
