# Supplementary material for: Stakeholder involvement in systematic reviews: a scoping review
Source: Syst Rev. 2018 Nov 24;7:208. doi: 10.1186/s13643-018-0852-0 (PMC6260873; doi:10.1186/s13643-018-0852-0)
Supplement: Supplementary file 6 — Table of excluded studies. (DOCX 50 kb) [file 13643_2018_852_MOESM6_ESM.docx]

**Additional File 6 – Table of Excluded studies**

| **Study author** | **year of publication** | **If not applicable, state reason why** |
| --- | --- | --- |
| Abelson | 2013 | Does not include a systematic review. |
| Adjagba | 2015 | This is a descriptive report of a web-based platform to support NITAGs, and does not relate to involvement. |
| Aggarwal | 2014 | This paper describes a systematic review (with no involvement) plus qualitative interviews. These two methods were carried out concurrently, with no overlap in the methods. The results were triangulated at the end of both studies (i.e. the results of one could not impact on the results of the other). |
| Al-Khatib | 2015 | Stakeholder involvement is relating to identification of research priorities only. |
| Allen | 2014 | Paper describes development of a resource (Evidence Aid) which brings together systematic reviews relating to response to disasters. Evidence Aid does not carry out the systematic reviews. Stakeholder involvement occurs to prioritise which systematic reviews to include in the resource. |
| Allsop | 2013 | No systematic review of literature is reported. |
| Andermann | 2016 | This paper is not a systematic review, and does not discuss involvement in any way. |
| Anderson | 2013 | Discussing complexity within systematic reviews, and is not focussed on involvement. |
| Anonymous, commentary on Bertholet | 2015 | Commentary is discussing user involvement in designing effective interventions. |
| Ardolino | 2012 | Consensus decision making relating to outcome measure selection |
| Arighi | 2011 | Does not include a systematic review. |
| Armstrong | 2013 | No involvement in this review. |
| Arnstein | 1969 | Describes ladder of citizen participation. Relevant background paper. |
| Austvoll-Dahlgren | 2015 | Not a systematic review of literature. |
| Aydin | 2015 | Not a report of a systematic review. |
| Bagley | 2016 | Does not describe involvement within a systematic review (focussed specifically on trials) |
| Baier | 2015 | States "we did not conduct a systematic review". |
| Barber | 2007 | Survey relating to consumer involvement in research, but no focus or information on systematic reviews. |
| Barham | 2011 | Describes involvement within NICE organisation |
| Bastian | 2011 | Focussed on methods for priority setting |
| Bastian | 1994 | Report relating to consumer involvement in Cochrane. Does not describe practical issues relating to involvement. |
| Baunack-Mayer | 2010 | Not directly related to systematic reviews (although systematic reviews were used to inform participants in relation to current evidence) |
| Belizan | 2010 | Does not describe involvement within a systematic review. |
| Bennetts | 2011 | Not related to systematic reviews. Consumer involvement in health care, not specific to research. |
| Bird | 2013 | Not related to systematic reviews. Discussion article. |
| Bjering | 2014 | No systematic review of literature is reported. |
| Bombard | 2011 | Involvement in HTA evaluation - this does not occur within the systematic review process (see Figure 1). MAS-OHTAC (Ontario Health Technology Advisory Committee; MAS, Medical Advisory Secretariat) review process is relevant background information - citation relevant as bibliography paper. |
| Bombard | 2013 | Involvement in HTA evaluation. |
| Boote | 2015 | Does not describe involvement in a SR. Is relevant for inclusion within bibliographic searching. |
| Boote | 2011 | Papers within this review are relevant for inclusion, and will be included. The review itself does not meet criteria for inclusion, although provides a useful background paper. |
| Booth | 2012 | Describes PROSPERO database. |
| Boulekdid | 2011 | Describes use of Delphi process for identifying quality indicators. |
| Braithwaite | 2010 | Not related to stakeholder involvement |
| Brouwers | 2010 | Describes the AGREE (Appraisal of Guidelines Research & Evaluation) tool |
| Brown | 2013 | Involvement occurred after completion of systematic review, and was aimed at gathering feedback on a guideline. |
| Brown | 2013 | Not related to stakeholder involvement |
| Brussoni | 2006 | Methods involve consulting completed systematic reviews, but not completion of systematic reviews. |
| Cairelli | 2013 | Not related to stakeholder involvement |
| Camp | 2014 | Systematic review was carried out independently to the expert panel meetings. |
| Caron-Flinterman | 2005 | Opinion/discussion paper, not specific to systematic reviews. Relevant background paper. |
| Cashman | 2008 | Discusses case examples of community-based participatory research (none specific to systematic reviews) |
| Chelmow | 2016 | Does not describe involvement, or describe a systematic review as part of the guideline. |
| Chen | 2010 | Describes processes relating to translation of papers to be included within reviews. |
| Cheung | 2016 | Describes development of principles and recommendations relating to patient involvement in outcomes research. Relevant background paper. |
| Christiaens | 2012 | Relates to involvement in health care policy decisions. |
| Clarke | 2010 | Relates to involvement in trials as a participant. |
| Cohen | 2013 | Not not relate to involvement in reviews, but to the processes involved in completing a review. |
| Concannon | 2012 | Introduces the 7Ps framework. Relevant background paper |
| Concannon | 2012 | Paper does not report a systematic review or study. Describes a taxonomy for describing stakeholders involved in research (including systematic reviews). |
| Costa | 2015 | This is not a systematic review, or a report of involvement in a systematic review. This is a report of involvement in expanding and evaluating a scale for assessing the methodological quality of systematic reviews. |
| Cottrell | 2015 | Describes results of research to identify benefits and challenges of stakeholder engagement in systematic reviews, based on a literature review and interviews. However does not describe methods of involving people in systematic reviews (relevant background paper) |
| Cottrell | 2014 | Report relating to stakeholder involvement in reviews, based on literature and interviews. |
| Crews | 2012 | Study is focussed on identification of priorities for future systematic reviews. |
| Crowley | 2012 | Prioritisation project. |
| Curtis | 2012 | Standards for patient engagement in outcomes research. |
| Danner | 2011 | Not focussed on a systematic review. Involvement in determining patient preferences. |
| Del Sorbo | 2015 | The expert panel which is referred to was for a previously reported study. There is no expert panel involvement reported for this review. |
| Deverka | 2012 | Discussion paper on stakeholder engagement in research (not specific to systematic reviews). |
| Dipankui | 2015 | Not a systematic review of literature. |
| Eichenauer | 2014 | No involvement reported in this review. |
| Evans | 2010 | No involvement in the systematic review. |
| Facey | 2010 | Not related to systematic reviews. |
| Faulkner | 2002 | Describes two examples of user-led research; neither are systematic reviews. |
| Fisher | 2010 | Systematic review was carried out before the involvement of the expert group. |
| Fitch | 2001 | This is a method of judgement applied after completion of a systematic review. |
| Fleurence | 2014 | Explores scores assigned to PCORI proposals, based on patient and stakeholder reviews |
| Flitcroft | 2011 | No systematic review of literature is reported. |
| Fong | 2014 | This paper is a review of existing guidelines (for melanoma). The methods of various guidelines are briefly discussed, including statements of whether consensus was reached by expert panel. But no further information is provided. |
| Forsythe | 2016 | Does not describe involvement in a SR. Is relevant for inclusion within bibliographic searching. |
| Foster | 2015 | No involvement is reported within the systematic review. |
| Fox | 2013 | No systematic review of literature is reported. |
| Frank | 2015 | This paper describes methods of involvement in outcomes research (not systematic reviews). The models presented arguably have some relevance (background paper). |
| Freyne | 2014 | No involvement in this review. Review is focussed on animal models, not humans. |
| Fudge | 2008 | An ethnographic study exploring user-involvement in stroke research (not systematic reviews) |
| Gagnon | 2011 | No involvement in the systematic review. |
| Gamble | 2014 | Describes involvement in a trial. |
| Gargon | 2014 | Review of methods of involvement in patient centred outcome research. Not directly relevant to systematic review methods, but does clearly identify the different methods of involvement which have been used in conjunction with systematic reviews to develop core outcome sets. SEE ALSO GORST 2016 - UPDATE OF THIS REVIEW |
| Gauvin | 2010 | Not related to systematic reviews |
| Gauvin | 2011 | Not related to systematic reviews. |
| Ghaumi | 2015 | This systematic review (which is focussed on participation in health promotion) does not have any stakeholder involvement. |
| Gillard | 2010 | Focus is on qualitative research, not systematic reviews. |
| Glenton | 2010 | This study explores how Cochrane review results can best be presented to a consumer audience. This is in general terms, ie.. Does not relate to involvement in a single review. |
| Gobat | 2015 | Does not appear to have been any involvement within this systematic review. |
| Gold | 2015 | This paper does report a systematic review, which relates to "consumer engagement". However this relates to involvement in health IT, not involvement in research. |
| Graham | 2006 | Paper is focused on knowledge translation. |
| Greene | 2011 | Involvement occurred after completion of systematic review, and was aimed at agreeing definitions, and not impacting on or interpreting the findings of the review. |
| Gregory | 2011 | No involvement reported in the review |
| Grimbizis | 2016 | Does not report a systematic review. |
| Grossberg | 2010 | No systematic review of literature is reported. |
| Guise | 2013 | This review is focussed on involvement in priority setting. Relevant background paper. |
| Guthrie | 2015 | Does not report a systematic review. Relevant background paper. |
| Hansen | 2011 | Describes how patient perspectives are reported within HTA reports; not including PPI |
| Hansen | 2011 | Focussed on methods of qualitative synthesis. |
| Harman | 2015 | Although this study included a systematic review, the study purpose related specifically to development of a core outcome set. There was no involvement within the review. |
| Hatemi | 2014 | Systematic review did not have any reported involvement. |
| Heikkinen | 2012 | No involvement is described within this systematic review. |
| Helfand | 2010 | Does not describe involvement in a SR. |
| Hesselink | 2013 | Focus is on involvement in reaching consensus over outcome measures. |
| Hillier | 2010 | Focussed on selection of outcome measures |
| Hivon | 2012 | Not focussed on involvement. |
| Hoffman | 2010 | Discusses good practice in involvement. Relevant background paper |
| Hoffman | 2010 | No systematic review of literature is reported. |
| Hofmann | 2014 | No systematic review of literature is reported. |
| Horey | 2010 | Focused on involvement at an organisational level, rather than individual review level. |
| Huisstede | 2014 | Although a systematic review had been carried out previously, this has been reported elsewhere, and did not have involvement. This paper reports on a consensus process only. |
| Husereau | 2014 | The authors state that (although literature was searched) no systematic review was carried out. |
| Inotai | 2012 | Does not report a systematic review of literature. |
| INVOLVE | 2012 | Supplement to briefing notes for researchers on public involvement in systematic reviews |
| Izquierdo | 2011 | Paper reports a systematic review and a qualitative study, but there was no involvement within the systematic review |
| Jain | 2014 | No systematic review of literature is reported. |
| Jaung | 2012 | This paper does not relate to healthcare, or have any healthcare related outcomes. |
| Johnson | 2009 | Describes development of a framework for health technology decisions. Not specific to involvement. |
| Jun | 2015 | These systematic reviews explore the evidence relating to the top research priorities of patients/carers, as identified in a previously reported JLA PSP. There was no further patient/carer involvement in these systematic reviews. |
| Kauffman | 2013 | Not related to systematic reviews. |
| Keown | 2008 | Focused on involvement at an organisational level, rather than individual review level. |
| Kho | 2010 | Focussed on recruitment to guideline study; no specific link to systematic review. |
| Klein | 2012 | Prioritisation project. |
| Kolasa | 2014 | Although this study included a systematic review and a survey, there was no involvement in the systematic review. |
| Kotter | 2013 | Systematic review of how patients are involved in quality indicator development. |
| Kreindler | 2009 | Not related to systematic reviews. |
| Kreis | 2013 | Focused on involvement at an organisational level, rather than individual review level. |
| Langlois | 2015 | A description of an Advisory Group on Health Systems Research Synthesis which brings together different collaborations, groups and institutions |
| Leeflang Marisk | 2015 | No stakeholder involvement reported. |
| Legare | 2011 | Systematic review of public involvement in guidelines |
| Lieberman | 2015 | No systematic review of literature is reported. |
| Likumahuwa-Ackman | 2015 | Does not include a systematic review. |
| Liu | 2011 | No involvement is reported within the systematic review. |
| Lossius | 2013 | Does not report a systematic review. |
| Lowes | 2011 | Provides a good description of stakeholder involvement, but this is in an intervention development study, and not specific to a systematic review. |
| MacMillan, M., Tarrant, M., Abraham, C., Morris, C. (2014). The association between children's contact with people with disabilities and their attitudes towards disability: a systematic review. Developmental Medicine and Child Neurology, 56(6), 529-546 | 2014 | No involvement is described within this systematic review |
| Manchikanti | 2014 | There is no involvement within the reported review of literature. |
| Marris | 2010 | Editorial. Not specific to involvement in systematic reviews. |
| Martin | 2012 | No systematic review of literature is reported. |
| Mavris | 2012 | No systematic review of literature is reported. |
| Mayer | 2012 | Editorial discussing importance of patients as research partners. Not specific to systematic reviews. |
| Mayer | 2015 | No involvement is reported within this systematic review. |
| McDonald | 2013 | No systematic review of literature is reported. |
| Menon | 2011 | Systematic review of involvement in HTA research, not related to systematic reviews |
| Mittler | 2013 | Focussed on consumer engagement in health care, not research (or systematic reviews) |
| Moffat | 2013 | Although a user-group considered literature, there was no systematic review. |
| Moran | 2011 | Focussed on public involvement in HTA program, and not specific to systematic reviews |
| Morris C, Shilling V, McHugh C, Wyatt K. (2011) Why it is crucial to involve families in all stages of childhood disability research. Developmental Medicine & Child Neurology, 53(8):769-71. | 2011 | Letter. Relates to involvement, but not specifically to systematic reviews. |
| Morrow | 2013 | No involvement is reported in this systematic review. |
| Mostovaya | 2014 | No involvement is reported within this systematic review. |
| Mullins | 2012 | Not specific to systematic reviews. |
| Nicholls | 2015 | No systematic review of literature is reported. |
| Norman | 2013 | Describes process of translating evidence-based recommendations into information; no involvement in the systematic review stage. |
| Oliver | 2008 | Does not describe methods of involvement in systematic reviews. Relevant background paper, including framework of public involvement in research. |
| Oliver | 2015 | Does not describe methods of involvement in systematic reviews. Relevant background paper, including framework of public involvement in research. |
| Oliver | 2004 | Focus is on priority setting, not systematic reviews. |
| Ortiz | 2012 | A systematic review was not carried out within this study. |
| Parekh | 2015 | Not a systematic review of literature. |
| Peckham | 2014 | This study did not include a systematic review of evidence. The study explored patient/public involvement in relation to local services and commissioning. |
| Perrier | 2015 | Not a systematic review. Pilot study for a RCT. |
| Petticrew | 2013 | Does not describe or discuss methods of involvement. |
| Piltch-Loeb | 2014 | This is not a systematic review, or a report of involvement in a systematic review. This is a report of the development of a 'peer assessment approach' for learning from public health emergencies. It does state that a 'literature review' was done, but no further details are provided. |
| Prictor | 2013 | Describes Cochrane Consumers & Communication Group. |
| Rees | 2012 | Although examples of involvement in systematic reviews are presented as examples, all of these are included individually in this review. |
| Ribeiro | 2010 | No systematic review of literature is reported. |
| Risso-Gill | 2015 | Does not report involvement within a systematic review. Reports a multi-method study which includes literature reviews and interviews. |
| Robinson | 2010 | Not a systematic literature review, but a search of website information. |
| Roque | 2014 | Does not report involvement within a systematic review. Reports a multi-method study which includes literature reviews and interviews. |
| Roseman | 2013 | Is focus on patient involvement in healthcare (not research); no involvement is reported in the systematic review. |
| Rosenbaum | 2010 | Does not describe involvement in a review, but involvement in developing a template for a summary of findings table, based on a completed review. |
| Rosenbaum | 2011 | Does not describe involvement in a review, but involvement in developing a template for a summary of findings table, based on a completed review. |
| Sainfort | 2013 | Involvement occurred after completion of the review, to add data/information, rather than be involved in the review findings. |
| Sanchez | 2014 | This is an analysis of RCT data, with no reported stakeholder involvement. |
| Sangster-Gormley | 2011 | No involvement is reported in this systematic review. |
| Sarasohn-Kahn 2013 |  | No involvement is described within this systematic review. |
| Sarrami-Foroush | 2015 | Does not report involvement in a systematic review. |
| Sarrami-Foroush | 2014 | Does not report involvement in a systematic review. |
| Schmittdiel | 2015 | No systematic review of literature is reported. |
| Schubart | 2011 | No involvement is reported in this systematic review. |
| SCIE | 2007 | The paper describes examples of user-involvement in reviews. Each example is included as a separate study. |
| Seale | 2014 | The investigator group for this systematic review comprised the researchers from primary studies - i.e. does not meet our definition of stakeholder involvement. |
| Serena | 2012 | No systematic review of literature is reported. |
| Shea | 2005 | Describes involvement in the Cochrane Musculoskeletal Group, but not within individual reviews. Relevant background paper |
| Singh | 2015 | This research builds on previously completed systematic reviews by other author groups. |
| Slutsky | 2010 | Introduction to methods guide for comparative effectiveness reviews. |
| South | 2016 | Reports a series of case-studies relating to involvement. One case study was a systematic review. This systematic review has been included. |
| Staniszewska | 2011 | The GRIPP checklist is a tool for reporting involvement in research. The is a relevant tool (although does not meet criteria for this systematic review). |
| Stansiszewska | 2011 | Describes the GRIPP checklist, for reporting PPI in research. Relevant background paper, but does not meet criteria for inclusion. |
| Swanson | 2011 | Not a healthcare related review / paper. |
| Tempfer | 2011 | Focus is on consumer participation in healthcare (not research). |
| Terry | 2012 | Focussed on user-involvement in teaching. No involvement reported in the systematic review. |
| Teunissen | 2013 | No involvement is reported in this systematic review. |
| Thompson | 2009 | Qualitative study describing researchers' attitudes towards involvement. Relevant background paper. |
| Towle | 2010 | No involvement is reported in this systematic review. |
| Tritter | 2006 | This paper is a critique of Arnstein's model of participation. |
| Trujillo-Martin | 2009 | No involvement reported |
| Truman | 2001 | Discusses involvement in health research. Not specific to systematic reviews. |
| Trusheim | 2014 | Does not include a systematic review. |
| Tuffrey-Wijne | 2010 | Describes involvement in qualitative analysis, not systematic reviews. |
| Turner | 2005 | Describes good practice in user-controlled research. Is not specific to systematic reviews. |
| Turner | 2010 | No discussion of involvement within guidelines. |
| Vale | 2012 | Focus in on involvement in primary research studies. |
| Vale | 2011a | Focus is on involvement in primary research studies. |
| van der Weijden | 2013 | Not related to systematic reviews. |
| van Est | 2011 | Not related to involvement in systematic reviews. |
| Wale | 2013 | Survey relating to how consumers would prioritise Cochrane reviews (as users of the reviews), rather than involvement of consumers in reviews. |
| Wallace | 2012 | Does not describe involvement in a systematic review. |
| Washington | 2011 | Describes PCORI (Patient-Centred Outcomes Research Institute). |
| Watt | 2011 | Did not report involvement in the systematic review |
| White | 2010 | No involvement is reported in this systematic review. |
| Whitlock | 2010 | Is focussed on prioritisation of reviews at an organisational level. |
| Whitty | 2013 | Survey relating to approaches of engaging the public in HTA, but no information specific to systematic reviews. |
| Willis | 2011 | Unable to access full text. Based on abstract only - no involvement is reported in this review. |
| Wilson | 2010 | No systematic review of literature is reported. |
| Wong | 2013 | Describes a Delphi approach to reach agreement on publication standards for realist reviews. |
| Wright | 2010 | Describes an international collaboration for participatory health research. Does not report on a systematic review. |
| Wright-Berryman | 2011 | No involvement is reported in this systematic review. |
| Yi | 2011 | Although a systematic review is reported, the involvement occurred independently of the review and not directly relating to the review. There was no involvement in the systematic review. |
| Young | 2012 | Although systematic reviews are referred to, they are not reported in this paper. |
| Zill | 2015 | Does not include a systematic review. |
| Zschocke | 2014 | This study combined a systematic review with qualitative interviews and focus groups, in order to develop and test a questionnaire. There was no user-involvement in the systematic review component. |
| Friele-Gutierre | 2014 | Reports involvement at an organisational level. Is not specific to systematic reviews. |
| Buhrlen | 2010 | Not a systematic review |

References available on request from authors.
